# Insights into epithelial cell senescence from transcriptome and secretome analysis of human oral keratinocytes

**DOI:** 10.18632/aging.202658

**Published:** 2021-02-12

**Authors:** Rachael E. Schwartz, Maxim N. Shokhirev, Leonardo R. Andrade, J. Silvio Gutkind, Ramiro Iglesias-Bartolome, Gerald S. Shadel

**Affiliations:** 1 Molecular and Cell Biology Laboratory, Salk Institute for Biological Studies and University of California – San Diego, La Jolla, CA 92037, USA; 2 Razavi Newman Integrative Genomics and Bioinformatics Core, Salk Institute for Biological Studies, La Jolla, CA 92037, USA; 3 Waitt Advanced Biophotonics Core, Salk Institute for Biological Studies, La Jolla, CA 92037, USA; 4 Department of Pharmacology and Moores Cancer Center, University of California – San Diego, La Jolla, CA 92093, USA; 5 Center for Cancer Research, National Cancer Institute, National Institutes of Health, Bethesda, MD 20892, USA

**Keywords:** cellular senescence, inflammation, extracellular vesicles, keratinocytes, carcinoma

## Abstract

Senescent cells produce chronic inflammation that contributes to the diseases and debilities of aging. How this process is orchestrated in epithelial cells, the origin of human carcinomas, is poorly understood. We used human normal oral keratinocytes (NOKs) to elucidate senescence programs in a prototype primary mucosal epithelial cell that senesces spontaneously. While NOKs exhibit several typical facets of senescence, they also display distinct characteristics. These include expression of p21WAF1/CIP1 at early passages, making this common marker of senescence unreliable in NOKs. Transcriptome analysis by RNA-seq revealed specific commonalities with and differences from cancer cells, explicating the tumor avoidance role of senescence. Repression of DNA repair genes that correlated with downregulation of E2F1 mRNA and protein was observed for two donors; a divergent result was seen for the third. Using proteomic profiling of soluble (non-vesicular) and extracellular vesicle (EV) associated secretions, we propose additions to the senescence associated secretory phenotype, including HSP60, which localizes to the surface of EVs. Finally, EVs from senescent NOKs activate interferon pathway signaling in THP-1 monocytes in a STING-dependent manner and associate with mitochondrial and nuclear DNA. Our results highlight senescence changes in epithelial cells and how they might contribute to chronic inflammation and age-related diseases.

## INTRODUCTION

Cancer, heart disease, neurodegenerative diseases, and stem cell dysfunction are major age-associated diseases and debilities with a substantial inflammatory component. Whereas transient inflammation serves productive purposes, such as eliminating pathogenic infections, chronic inflammation is detrimental. Senescent cells, a troubling source of unresolved inflammation during aging, have been found in numerous organs, including brain (astrocytes, microglia) [1], skin (fibroblasts, melanocytes, epithelial cells) [2], the cardiovascular system (endothelial cells) [3] and skeletal muscle (satellite/stem cells) [4].

Senescence is triggered by irreparable DNA damage and serves as a cancer avoidance mechanism by halting the growth of cells with unstable genomes, which can become malignant. It is characterized by replicative arrest and a pro-inflammatory senescence associated secretory phenotype (SASP) [5]. Because the immune system cannot always eliminate senescent cells [6], they persist as the organism ages [7]. While a foundational understanding of cellular senescence is provided by the excellent work already done, primarily in fibroblasts [5, 8], different cell types display variability in senescence. An understanding of each of them is essential to development of properly targeted therapeutics [9]. Given that senescence serves as a defense against cancer, and most cancers are carcinomas, which arise in epithelial tissue, understanding of epithelial cell senescence is especially important. Thus, we profiled senescence in normal oral keratinocytes (NOKs). NOKs senesce spontaneously over a short time in culture at atmospheric oxygen; however, individual cells in the population senesce at different rates. This allowed us to characterize senescence as cells at different stages of the process interact, which is more physiologically relevant than the common approach of inducing senescence simultaneously in an entire population (*e.g.*, by irradiation, chemical treatment, or oncogenic transformation). While a few gene expression profiles of senescence in NOKs have been reported [10–13], those works were necessarily constrained by limitations associated with the microarray technology that was available at the time.

The SASP has emerged as an important feature of senescent cells. It has been defined primarily in terms of secreted soluble proteins, some of which vary between cell types [5]. Far less is understood about the contribution of vesicles released by senescent cells, which we undertook to measure. EVs in senescence include microvesicles and exosomes. Microvesicles (100 to 1,000 nm diameter) bud off the plasma membrane. Exosomes (30 to 150 nm diameter) are formed in multi-vesicular bodies (MVBs) via the endocytic pathway and are released when the MVB merges with the plasma membrane. Major functions of EVs include disposal of unwanted cellular contents, signaling through receptors on target cells, and transfer of cellular contents between cells [14]. Here, we analyze the senescing NOK secretome for the first time.

## RESULTS

### NOKs display increases in established indicia of senescence, excepting p21WAF1/CIP1

**Figure 1:**
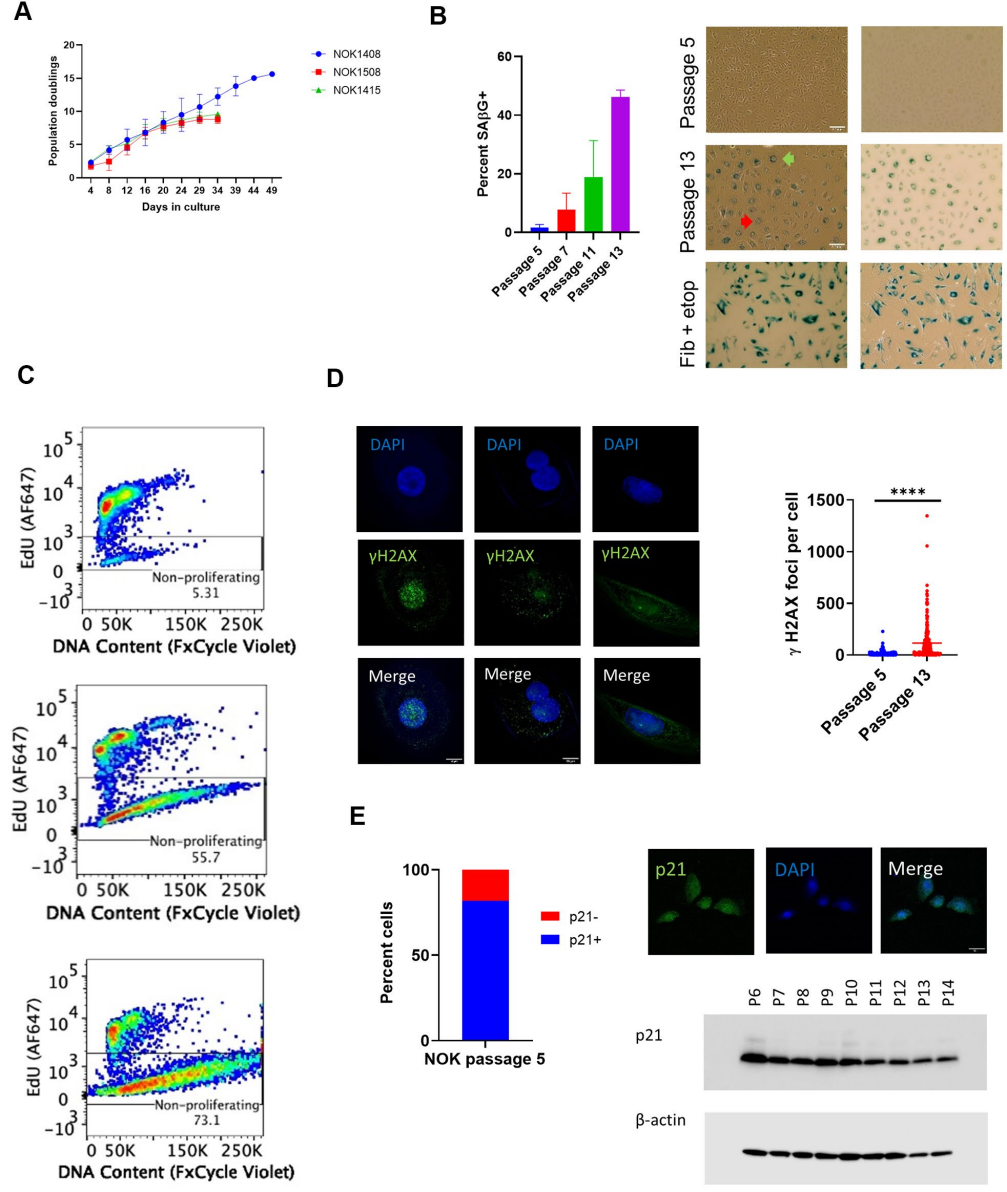
**Indicia of senescence.** (**A**) Population doubling. Y-axis is cumulative population doubling (PD) and X-axis is days in culture starting with passage 5. (Mean of 2 replicates per donor ± SD.) Cumulative PD = 3.322 x [log N(t) – log N(0)] + X, where N(0) is number of cells plated (5 x 10^5^), N(t) is number of cells at next passage, and X is PD for prior passages. (**B**) SAβG+ cells increased with passaging. (Top, passage 5; middle, passage 13, Donor 1415). (Mean ± SD.) Positive control (bottom) is etoposide-treated BJ fibroblasts. (Left, phase contrast; right, brightfield. 20x magnification.) Multinucleate cell, red arrow. Perinuclear vacuoles, green arrow. (**C**) Representative EdU flow cytometry results. (Donor 1415. Top, passage 5; middle, passage 10; bottom, last passage). (**D**) γH2AX IF. Staining shows significant increase (p < 0.0001, two-tailed t-test) in DSBs with passaging (Donors 1415 and 1408, combined), and binucleation and cytosolic foci in passage 13 cells (Donor 1415, left and center column images; Donor 1408, right column images). (**E**) Cells positive for p21WAF1/CIP1. Percent positive in nucleus at passage 5 (640 total cells from Donors 1408 and 1415). IF shows nuclear p21WAF1/CIP1 protein in passage 5 cells (Donor 1408). Immunoblot shows decline in p21WAF1/CIP1.

We measured cell population growth for six replicates, two from each of 3 donors (NOK1408 and 1508 were females, aged 18 and 20 years, respectively, and NOK1415 was a male, aged 35 years), starting at passage 5. Each replicate attained zero population growth, and therefore ceased population doubling, between passages 13 and 16 (Figure 1A). Positive staining for senescence-associated β-galactosidase (SAβG), which we performed for four replicates (Donors 1408 and 1415), increased with passaging (Figure 1B). Morphological changes included enlarged cell size and development of perinuclear vacuoles (Figure 1B).

Flow cytometry for incorporation of the thymidine analog 5-ethynyl-2 deoxyuridine (EdU) into nuclear DNA, a measure of cell proliferation, revealed a decline in cells staining positive over time. All three donors showed a balance of proliferating and non-proliferating cells at passage 10 (Figure 1C and Supplementary Figure 1A). Analysis of DNA content showed a loss by senescent NOKs of a distinct cell population with the normal diploid content that was seen with proliferating cells (Figure 1C and Supplementary Figure 1A). This suggests that a portion of NOKs were arrested in a multinucleate state and/or experienced nuclear blebbing of chromatin fragments into the cytoplasm, both of which are features of senescence [15]. Staining for phosphorylated histone H2AX (γH2AX) showed such multinucleate cells and both nuclear and cytoplasmic γH2AX at passage 13 (Figure 1D and Supplementary Figure 1B). Quantification of combined nuclear and cytoplasmic γH2AX foci demonstrated a significant increase in unrepaired DNA double-strand breaks (DSBs) accompanying senescence (Figure 1D).

Senescent cell cycle arrest can be mediated by the p16INK4A or p21WAF1/CIP1 cyclin-dependent kinase inhibitors. Quantified immunofluorescence (IF) for p16 significantly increased at passage 13 compared to passage 5 (Supplementary Figure 1C). While p21 has long been considered a sign of senescence in fibroblasts [16], we found that 82% of cells stained positive for nuclear p21 at passage 5 (Figure 1E and Supplementary Figure 1D, 1E). Using a different p21 antibody, we performed an immunoblot of whole cell lysate, confirming that p21 was present at early passages and showing that it decreased over time (Figure 1E and Supplementary Figure 1F).

In summary, we established that NOKs that senesce without irradiation, chemical treatment, or transfection with an oncogene satisfy several criteria of senescence. Some of the assays we used, such as measuring population growth and SAβG positivity, have previously been presented as evidence of senescence in NOKs [10–13]. However, our analysis of spontaneous senescence was more comprehensive (*e.g.*, EdU and DNA content flow cytometry, quantification of γH2AX foci). Our data also indicate that p21 is not a trustworthy marker of senescence in these cells.

### Unbiased, global RNA-sequencing of NOK senescence

We performed total RNA-seq for each replicate at passage 5, passage 10, and the final passage (Supplementary Tables 1, 2). Expression of 6,173 genes was significantly changed (p < 0.05) when comparing transcript levels at the final passage to passage 5. Principal component analysis (PCA) (Supplementary Figure 4A) showed substantial variability among the gene expression patterns of the three donors at passage 5, when most of the cells are actively proliferating. At passage 5, the two female donors were more alike than the third, male donor. However, all of the samples at final passage clustered together and away from the passage 5 samples. This convergence at the final passage, where senescent cells had accumulated, suggests commonality, but not complete identity, in the senescence gene expression signature.

**Figure 2:**
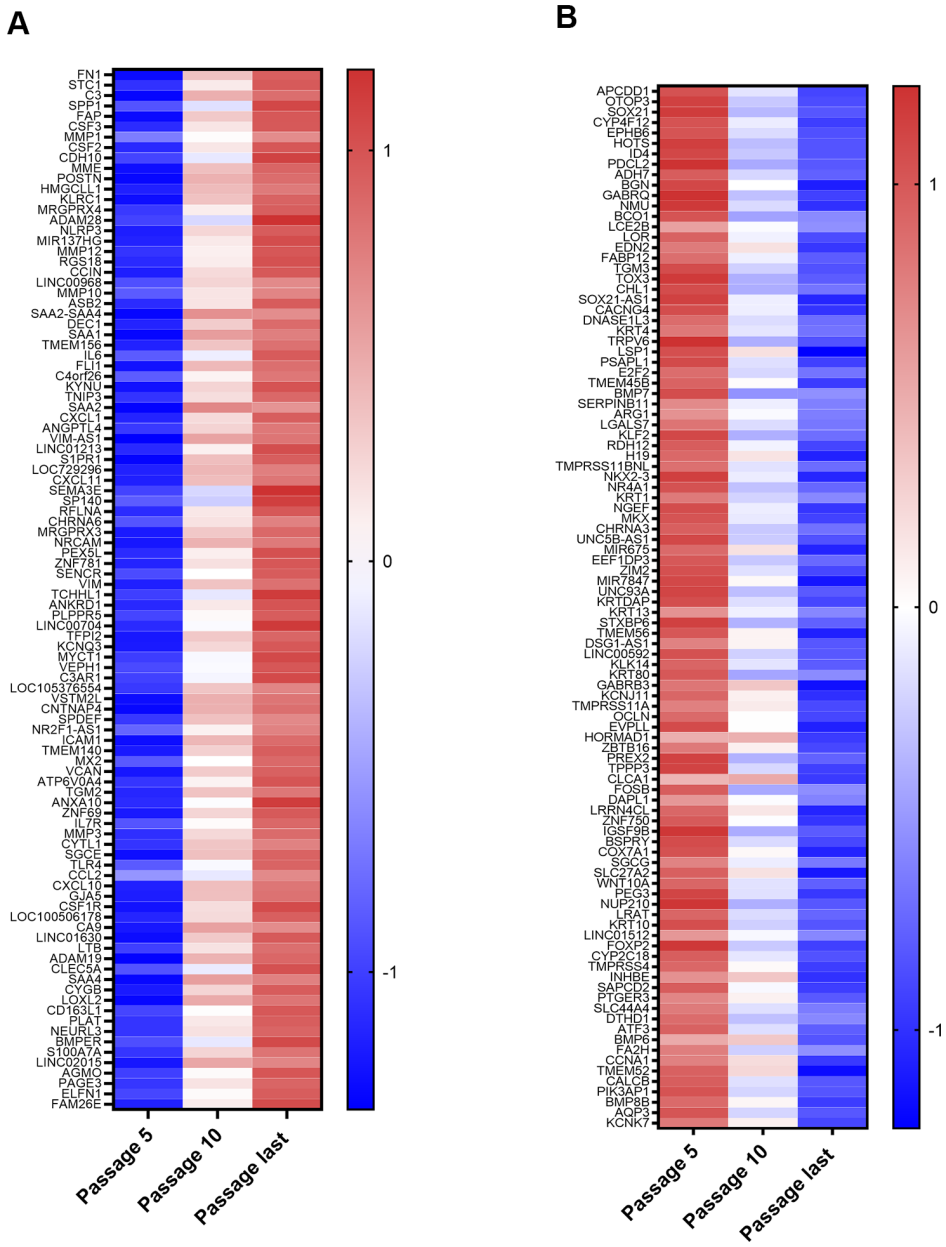
**Top 100 Upregulated and Downregulated Genes.** (**A**) Top 100 upregulated genes. These include those coding for proteins with functions in inflammation, proteases, protease inhibitors, and genes implicated in cancer. (**B**) Top 100 downregulated genes. These include 52 genes upregulated in at least one type of cancer (*ID4, PDCL2, ADH7, BGN, GABRQ, NMU, LCE2B, LOR, FABP12, CACNG4, KRT4, TRPV6, TMEM45B, SERPINB11, ARG1, LGALS7, NKX2-3, NR4A1, KRT1, NGEF, UNC5B-AS1, MIR675, KRTDAP, KRT13, TMEM56, LINC00592, KLK14, KRT80, TMPRSS11A, OCLN, HORMAD1, PREX2, TPPP3, CLCA1, FOSB, DAPL1, LRRN4CL, IGSF9B, SLC27A2, WNT10A, NUP210, KRT10, LINC01512, CYP2C18, TMPRSS4, INHBE, SAPCD2, PTGER3, SLC44A4, CALCB, PIK3AP1, AQP3*).

The top 100 up and down differentially expressed genes from passage 5 to the last passage were identified. After a stringent p-value cut-off (p < 0.01) was applied, the 100 genes with the greatest fold changes were then selected (Figure 2A, 2B). The list of top 100 upregulated mRNAs included genes involved in promotion of inflammatory processes (*C3, SPP1, CSF2, CSF3, IL6, NLRP3, CXCL1, CXCL11, S100A7A, CSF1R, C3AR1, TLR4, CCL2, CXCL10, CSFR1, LTB*) or responsive to inflammatory conditions (*FAP, SAA1, SAA2, SAA4, SAA2-4, ANKRD1, MX2*). Others limit inflammation (*TNIP3, SP140*). There were also several proteases (*MME, MMP1, MMP12, MMP10, MMP3, PLAT, ADAM19*) and a protease inhibitor (*TFP12*). Genes whose upregulation is associated with some cancers (*TMEM140, ANXA10, ZNF69, CA9, LOXL2, PAGE3, ELFN1*) were also on this list, as was a putative tumor suppressor (*DEC1*). The dominant theme among the downregulated genes was silencing of genes whose products are upregulated in various cancers. Of the 100 genes, 52 (including some transcribed to non-coding RNAs) have been identified as upregulated in at least one type of cancer (Figure 2B). Mixed results have been found for another 10 (*HOTS, EDN2, TGM3, CHL1, SOX21-AS1, ZBTB16, PEG3, ATF3, CCNA1, KCNK7*), which are upregulated in some cancers, but downregulated in others. A third group of downregulated genes includes tumor suppressors and transcription factors for tumor suppressors that are lost or mutated in cancer (*EPHB6, E2F2, KLF2, H19, ZNF750, COX7A1, FOXP2, BMP6, FA2H, BMP8B*). This included *COX7A1*, a subunit of cytochrome c oxidase, complex IV of the mitochondrial electron transport chain, suggesting possible downregulation of mitochondrial respiration during NOK senescence.

We performed Gene Ontology (GO) overrepresentation analysis using WebGestalt. This revealed that senescence brings upregulation of inflammatory pathways, including responses to IFN, and downregulation of peptide cross-linking, which is needed to stabilize proper protein folding (Supplementary Figure 2A). Interim comparisons show similar pathway changes, with the exception that acute inflammatory response was downregulated at last passage compared to passage 10 (Supplementary Figure 2B, 2C). The seven acute inflammatory response genes that were downregulated for this interim comparison (*NUPR1, ADAM8, CFB, CXCR2, TREM1, TNFSF11, PRGER3*), however, were largely different from the inflammatory genes that were upregulated when comparing the last passage to passage 5.

We used HOMER (Hypergeometric Optimization of Motif EnRichment) *de novo* motif enrichment software to identify the transcription factors most likely responsible for the changes in mRNA observed with senescence. HOMER identifies known transcription factor binding motifs in the promoter regions of the genes from which those mRNAs are transcribed. We examined genes that were significantly changed (p < 0.05) up or down at passage 10 vs passage 5, last passage vs passage 5, and last passage vs passage 10. We thereby sought to distinguish changes that occurred along the entire process of senescence from those that were limited to a period when cells were mostly proliferating or senescent. Applying an FDR of 0.05, three of these six searches produced results: up in last passage vs passage 5, down in last passage vs passage 5, and down in passage 10 vs passage 5 (Supplementary Figure 3). Comparing the last passage to passage 5, HOMER identified 3 motifs among the upregulated genes and 5 motifs among the downregulated genes. Promoters of genes that were upregulated were enriched in binding sites for NF-κB and the IFN stimulated response element (ISRE) (Supplementary Figure 3A). Of the five motifs in downregulated genes, the top hit was for Smad3 (Supplementary Figure 3B). Smad3 activity is negatively regulated by cyclin D1 [17]; an increase in cyclin D1 is a hallmark of senescence and was seen in senescent NOKs (Supplementary Table 1). Some motifs appeared only when comparing genes downregulated at passage 10 versus passage 5 (Supplementary Figure 3C). One was the CCAAT-box. Among the transcription factors binding this motif is nuclear factor Y, whose target genes are involved in cell cycle progression and DNA repair [18]. Two of the motifs are rich in guanine bases, which are susceptible to reactive oxygen species-induced 8-oxoG DNA lesions that may hinder transcription factor binding [19].

There was also a change in mRNA for some transcription factors that bind these motifs. Comparing the last passage to passage 5, mRNA was significantly upregulated for *STAT1, STAT2*, and *IRF9* (Supplementary Table 1), which bind the ISRE. Conversely, *MEF2A* and *KLF4* mRNA were significantly downregulated from passage 5 to passage 10 (Supplementary Table 1). The fact that mRNA for many of the transcription factors whose binding motifs were identified by HOMER was largely unchanged suggests that the abundance and/or activity of these factors is regulated at a different level. For example, activity of the NF-κB subunit p65 is known to depend on destruction of its inhibitor, IκB, and on subsequent phosphorylation and acetylation of p65.

**Figure 3:**
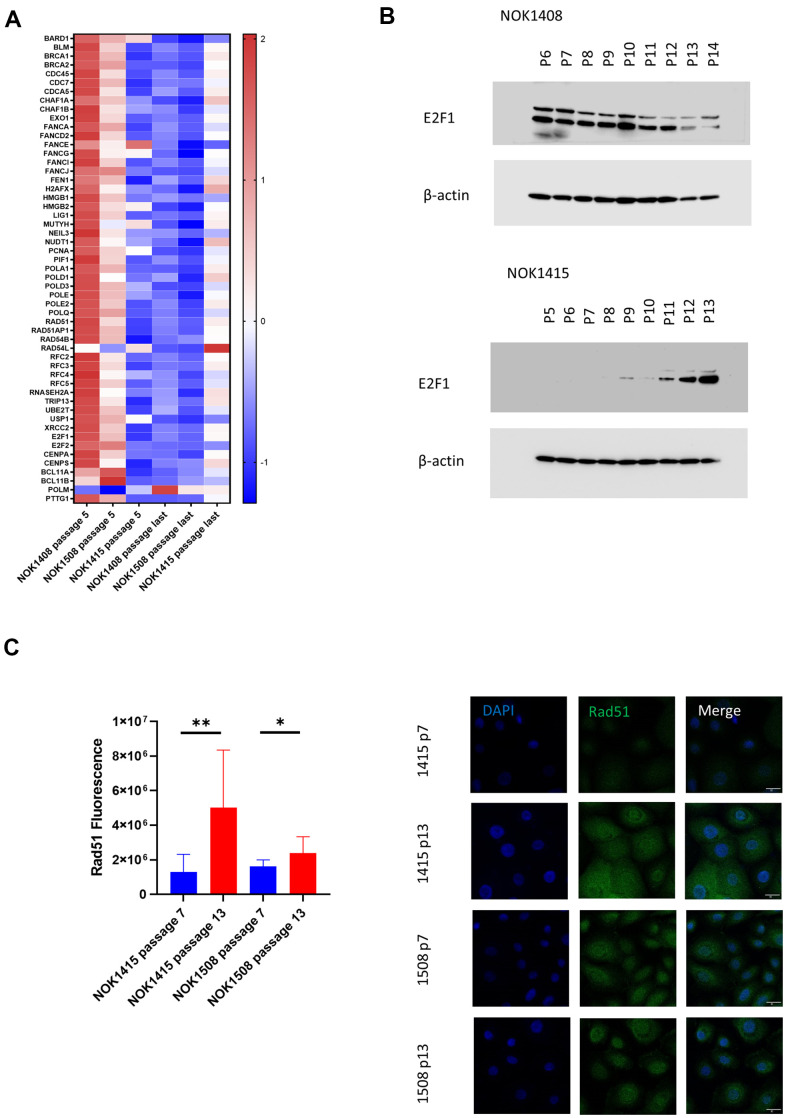
**Unbiased, global RNA-sequencing.** (**A**) Heat map by donor of DNA repair genes. Z-scores for genes involved in DNA repair whose mRNA was significantly downregulated (adj. p-value < 0.05 comparing last passage to passage 5) for the two female donors plus polymerase μ (second from bottom), which was significantly upregulated. (**B**) Immunoblotting for E2F1. Protein decreases with senescence for a female donor (Donor 1408), but increases for male donor (Donor 1415). (**C**) Change with senescence in Rad51 IF. Quantification (left, p-values determined using two-tailed t-test) and representative images (right).

Since downregulation of DNA repair genes has previously been observed in senescence [20], we were surprised to find only a weak trend toward downregulation of these genes in our dataset. When we broke down the data by donor, we saw that elements of several DNA repair mechanisms were significantly down for the two female donors (Donors 1408 and 1508) (Figure 3A), but the pattern was in the opposite direction for the male donor (Donor 1415) (Figure 3A). There was, however, an increase in mRNA for DNA polymerase mu (*POLM*), which participates in non-homologous end-joining (NHEJ), for all three donors (Figure 3A). E2F1, a key transcription factor in DNA repair [20], was one of the genes demonstrating the divergent pattern at the mRNA level (Supplementary Table 1). Corresponding with the mRNA, we found that the amount of E2F1 protein decreased with senescence for one of the female donors (Figure 3B and Supplementary Figure 4B), but increased with senescence for the male donor (Figure 3B and Supplementary Figure 4C). The multiple bands on the blots are likely due to alternatively spliced versions of the protein [21]. We also performed IF staining of cells for Rad51, the protein product of another DNA repair gene that showed the divergent, donor-specific pattern at the mRNA level (Figure 3C). Rad51 mediates the repair of DNA DSBs by facilitating homologous recombination [22]. Although average fluorescence per cell increased with senescence for both the male (NOK1415) and female (NOK1508) donors, the increase was much stronger for the male donor compared to the female donor. Rad51 fluorescence increased four-fold for the male donor, but only 50% for the female donor, comparing passage 13 to passage 7 (Figure 3C). The greater increase in protein for the male donor is consistent with the increase in *RAD51* mRNA for that donor. The results for the female donor suggest that post-transcriptional mechanisms lead to some increase in the amount of Rad51 protein, despite downregulation of the mRNA.

We compared our RNA-seq results to those from a study of various cell types induced to senesce by DNA damage, telomere shortening, and oncogenic transformation, in order to extract a core signature of senescence [2]. That study identified 55 genes significantly changed at the mRNA level in each cell type examined and for each method by which senescence was induced. Our list of genes significantly changed with NOK senescence showed an overlap with this core signature of 29 genes: 16 genes (*ADPGK, B4GALT7, CCND1, DDA1, DGKA, FAM214B, GDNF, P4HA2, PDLIM4, PLXNA3, POFUT2, RAI14, SLC16A3, TAF13, TMEM87B, ZNHIT1*) were significantly upregulated in both data sets, 12 genes (*ARHGAP35, ARID2, C2CD5, CREBBP, MEIS1, NFIA, PCIF1, RHNO1, SPATA6, SPIN4, STAG1, USP6NL*) were significantly downregulated in both, and one (*BCL2L2*) was up in the core signature but down in NOKs.

These data thus establish several fundamental aspects of NOK senescence. They are consistent with the characterization of senescence as a cancer avoidance mechanism, although one that is not always fully successful. NOKs from different donors display senescence profiles that are alike in important respects (*e.g.*, upregulation of inflammation), but that differ with regard to DNA repair gene expression. While some senescence-associated changes occur continuously as the cell population senesces, others are predominant when the population is largely proliferating but beginning to senesce. Moreover, whereas senescence in NOKs bears a noticeable resemblance to senescence in other cell types, it fails to display some changes previously identified as core, and, for one gene, the change with senescence is in the opposite direction of the core signature.

### Unbiased, global mass spectrometry of conditioned medium and extracellular vesicles

**Figure 4:**
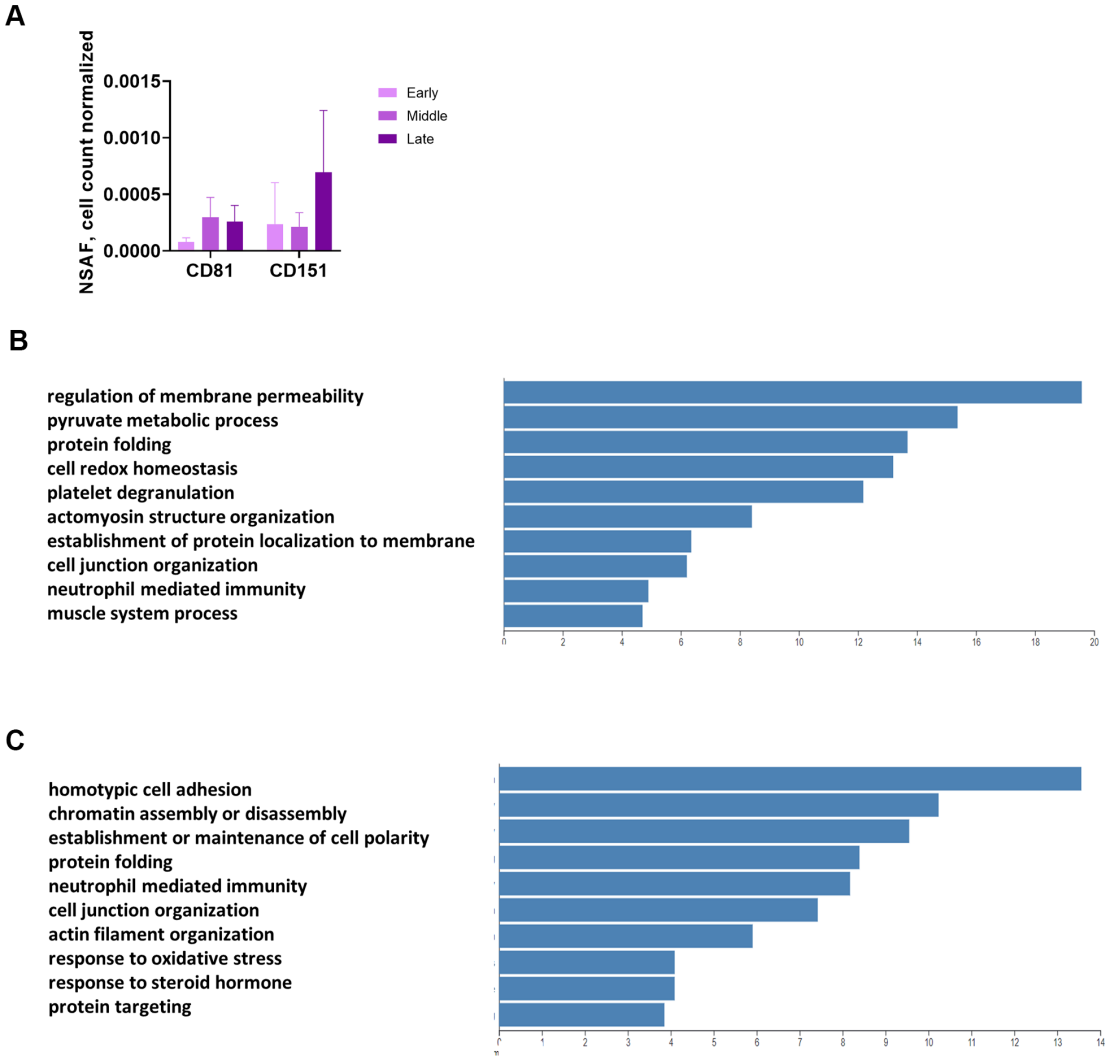
**Unbiased, global mass spectrometry of CM and EVs.** (**A**) Mass spectrometry of proteins isolated from CM by UC shows enrichment for exosomal markers. Mass spectrometry results are expressed as NSAF per 10^6^ cells (spectral abundance factor normalized for protein size and cell count). (**B**) Overrepresentation analysis of proteins significantly increased in CM with senescence. Y-axis shows enrichment ratio. (**C**) Overrepresentation analysis of proteins significantly increased in EV pellet with senescence. Y-axis shows enrichment ratio. (**B**, **C**, p < 0.01 comparing late to early and mid to early).

We used mass spectrometry to conduct an unbiased analysis of conditioned medium (CM) and proteins enriched in EVs from CM (2 replicates from each of Donors 1508 and 1415, 1 replicate from Donor 1408) at 3 time points. The EV enrichment protocol uses differential centrifugation, filtering, and ultracentrifugation (UC), yet some non-vesicular proteins remain in the UC pellet. Prior to filtering, we set aside a sample of CM for analysis. Mass spectrometric analysis identified approximately 2,500 proteins that were present in at least one of the thirty (CM and EV for each of the 5 replicates at 3 time points) samples (Supplementary Table 3). Two known surface markers of exosomes, CD81 and CD151, were present in the UC pellet, although they were not plentiful enough to be found in the correlating CM samples, showing that we effectively enriched for EVs (Figure 4A and Supplementary Table 3). The UC product is hereafter referred to as “the EV pellet.”

Overrepresentation analysis of the CM and EV proteins revealed some overlap in the operative pathways for the proteins secreted in soluble versus vesicular form, but several differences as well. We identified proteins with strong differential expression (p < 0.01) from both the early to late and mid to late passages (Supplementary Tables 4, 5). None of the proteins were differentially expressed with this level of significance from early to mid passages. Protein folding was an important pathway for proteins released by both routes, as heat shock proteins and isomerases were increased with senescence in both the CM (Figure 4B) and EV fractions (Figure 4C). However, the top 2 functions for CM were regulation of membrane permeability and pyruvate metabolic process (Figure 4B), whereas the top 2 for EV were homotypic cell-cell adhesion and chromatin assembly or disassembly (Figure 4C).

We also analyzed the mass spec results from CM and the EV pellet for significant (p < 0.05) changes, comparing each of the three stages (Supplementary Tables 4, 5). The top five proteins for five of the six comparisons changed upward (Table 1A–1E). The exception was changes in the EV pellet from early stage to mid stage, for which two proteins, components of the 60S ribosomal subunit (L36a and L36a-like), declined significantly (Table 1F). As the software can attribute the same spectral counts to two proteins that contain the same peptides, and these two proteins are similar, only one of them may actually have changed significantly.

**Table 1:** Changes in proteins secreted with senescence progression.

**A.** **CM** **Late vs Early**	**Gene**	**Name**	**Direction**
	*S100A6*	Protein S100-A6	Up
	*HSP90AA2P*	Heat shock protein HSP 90-alpha A2	Up
	*TPI1*	Triosephosphate isomerase	Up
	*HSP90AB2P*	Putative heat shock protein HSP 90-beta 2	Up
	*SH3BGRL3*	SH3 domain-binding glutamic acid-rich-like protein 3	Up
**B.** **EV** **Late vs Early**	**Gene**	**Name**	**Direction**
	*S100A2*	Protein S100-A2	Up
	*MVP*	Major vault protein	Up
	*SERPINH1*	Serpin H1	Up
	*UBE2V2*	Ubiquitin-conjugating enzyme E2 variant 2	Up
	*GSN*	Gelsolin	Up
**C.** **CM** **Late vs Mid**	**Gene**	**Name**	**Direction**
	*TPI1*	Triosephosphate isomerase	Up
	*HSP90AA2P*	Heat shock protein HSP 90-alpha A2	Up
	*HSP90AB2P*	Putative heat shock protein HSP 90-beta 2	Up
	*S100A6*	Protein S100-A6	Up
	*SH3BGRL3*	SH3 domain-binding glutamic acid-rich-like protein 3	Up
**D.** **EV** **Late vs Mid**	**Gene**	**Name**	**Direction**
	*S100A2*	Protein S100-A2	Up
	*MVP*	Major vault protein	Up
	*GSN*	Gelsolin	Up
	*UFM1*	Ubiquitin-fold modifier	Up
	*SERPINH1*	Serpin H1	Up
**E.** **CM** **Mid vs Early**	**Gene**	**Name**	**Direction**
	*LGAL53BP*	Galectin-3-binding protein	Up
	*APP*	Amyloid-beta precursor protein	Up
	*IGFBP7*	Insulin-like growth factor-binding protein 7	Up
	*TF*	Serotransferrin	Up
	*AGRN*	Agrin	Up
**F.** **EV** **Mid vs Early**	**Gene**	**Name**	**Direction**
	*RPL36A*	60S ribosomal protein L36a	Down
	*RPL36AL*	60S ribosomal protein L36a-like	Down

Top 5 significantly changed (p < 0.05) proteins in CM and EV pellet when comparing early, middle, and late passages. Only 2 proteins were significantly changed in the EV pellet when comparing mid to early passages.

There was an overlap between proteins increasing from early to late and those increasing from mid to late (Table 1A–1D), suggesting an uninterrupted increase in secretion of these proteins as the percentage of senescent cells in the population grew. The most significantly (lowest adjusted p-value) changed proteins from early to late for CM and EVs are two members of the S100A family of calcium-binding proteins: S100A6 for CM and S100A2 for the EV pellet. S100A molecules are damage-associated molecular patterns (DAMPs) that promote inflammation [23]. Another top increased protein in CM, HSP90, has previously been found in both vesicular and non-vesicular fractions [24] and is also a DAMP [25]. Triosephosphate isomerase, an enzyme of glycolysis, may be ejected by NOKs to subvert reliance on the glycolytic metabolism employed by cancer cells to survive and proliferate (*i.e.*, the Warburg effect). Major vault protein accounts for the largest portion of the mass of vaults, which are cytoplasmic, non-vesicular ribonucleoprotein structures that can be released by cells [24]. Ubiquitin-fold modifier 1 is a ubiquitin-like molecule. Its addition to a protein may play a role in its being sorted into an EV [26]. The only top five protein also present in clean medium is serotransferrin. Its increase in CM with passaging suggests that NOKs may take up less of it as they senesce.

These data provide an overview of the secretory aspect of NOK senescence that emphasizes the pro-inflammatory nature of both the soluble and vesicular components and the secretion of proteins associated with proteostasis. Simultaneously, the data highlight a significant decrease in release of a 60S ribosomal protein that occurs in the earlier phase of senescence (Table 1F).

### Inflammatory pathways are upregulated in NOK senescence

The SASP encompasses inflammatory components, factors involved in transmitting senescence to other cells and altering the environment in a manner that facilitates tumorigenesis, and regulators of these elements. The most comprehensive single published list of these components catalogued approximately 70 proteins [5].

**Figure 5:**
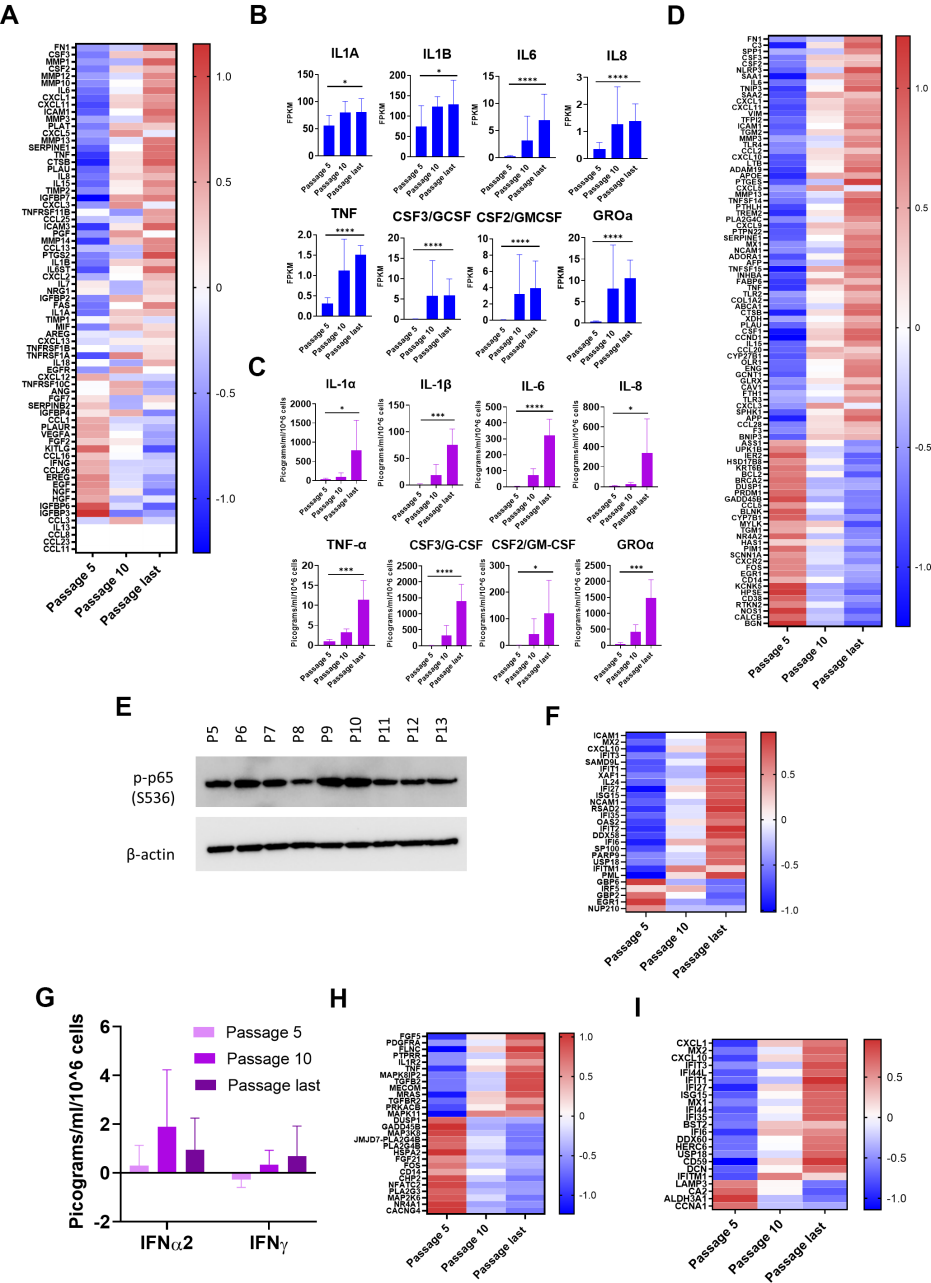
**Inflammatory pathways upregulated with senescence.** (**A**) Heat map of SASP protein components. Z-scores of changes in mRNA show upregulation, downregulation, or little change. Completely white rows represent zero FPKM at all passages for all donors. (**B**) mRNA levels of 8 selected major inflammatory SASP elements. Increases from passage 5 to last passage were significant (p < 0.05) (Mean ± SD). Y-axis scales differ. (**C**) Protein levels of the same 8 major inflammatory SASP elements in CM. Multiplex immunoassay results (Mean ± SD). Results normalized by cell count and by subtracting amounts of these proteins contained in the same quantity of clean medium. Y-axis scales differ. (Significance determined by t-tests.) (**D**) Heat map of differentially expressed genes (DEGs) in NF-κB pathways. Z-scores of mRNA changes. DEGs defined as adjusted p-value of change from passage 5 to last passage was < 0.05 and log2-fold change >1.5 or less than -1.5. (**E**) Immunoblot for p-p65 (S536). Indication of NF-κB transcriptional activity (Donor 1415). (**F**) Heat map of DEGs in IFN pathways. Z-scores of mRNA changes. DEGs defined as in Figure 5D. (**G**) IFN levels in CM are low at all stages. Determined and normalized as in Figure 5C. None of the changes were significant (p < 0.05). (**H**) Heat map of DEGs in p38MAPK pathways. Z-scores of mRNA changes. DEGs defined as in Figure 5D. (**I**) Heat map of IRDS DEGs. Z-scores of mRNA changes in IFN pro-survival gene set. DEGs defined as in Figure 5D. For Figure 5B, 5C, *p<0.05, **p<0.01, ***p<0.001, ****p<0.0001.

Our RNA-seq data showed a strong (p < 0.05 and log2 fold increase > 1.5) increase in mRNA for 23 SASP components, while 3 components were similarly decreased, and 4 were not present (Figure 5A, Table 2). The increases in mRNA were found in all SASP categories, including inflammatory factors, growth factors and their regulators, proteases, and protease inhibitors (Figure 5A). We confirmed increases for five SASP components with RT-qPCR (Supplementary Figure 5A). Comparison of mRNA levels at passages 5, 10, and final for eight major inflammatory SASP elements shows significant upregulation (Figure 5B).

**Table 2:** SASP elements significantly changed at mRNA level.

	**SASP DEGs**
Upward change	*FN1, CSF3, MMP1, CSF2, MMP12, MMP10, IL6, CXCL1, CXCL11, ICAM1, MMP3, PLAT, CXCL5, MMP13, SERPINE1, TNF, CTSB, PLAU, IL8, IL15, TIMP2, IGFBP7, CXCL3*
Downward change	*HGF, IGFBP6, IGFBP3*

26 genes for SASP components were differentially expressed (p < 0.05 and log2 fold increase > 1.5 or decrease < -1.5), comparing the last passage to passage 5.

Analysis of proteins in CM shows functionality of the secretory aspect of the SASP in NOKs. We performed a multiplex immunoassay and found significant increases in the same eight selected major inflammatory factors (Figure 5C). Notably, IL-1α, which is both an inducer and target of NF-κB transcription, was secreted by NOKs in increasing amounts as cells senesced, although the quantity varied between samples (from 74.68 to 1888.2553 pg/ml/10^6^ cells at the final passage). IL-1α is displayed on the surface of senescent fibroblasts, but little is secreted by those cells [27]. Other SASP proteins were present in CM in increasing amounts, despite the fact that changes in their mRNA levels were not significant (other than IL-15) (Supplementary Figure 5B). Except for VEGFA, the amounts were small. Secretion of EGF increased with senescence, despite the significant decrease in mRNA.

Proteomic analysis found that five SASP components increased significantly (p < 0.05) in the EV pellet with senescence: SERPINE1, cathepsin B, IL-18, TIMP1, and EGFR (Supplementary Table 5). Notably, IL-18 mRNA did not significantly change (Supplementary Table 1). This suggests that EVs may play a greater role in perpetrating chronic inflammation via the SASP than has been appreciated previously.

Three major inflammatory pathways have been found to be upregulated in senescence: NF-κB, cGAS-STING (cyclic GMP-AMP synthase-stimulator of interferon genes), and p38MAPK. Initially, NF-κB is activated as part of the DNA damage response [28]. Nuclear or mitochondrial DNA (mtDNA) in the cytosol is detected by cGAS; this triggers the STING pathway, which activates the IFN and NF-κB signaling pathways [15, 29]. Activation of p38MAPK in senescent cells has also been found to increase NF-κB transcriptional activity [30]. Our RNA-seq results showed strong upregulation of many elements of the NF-κB pathway (Figure 5D). Upregulated genes include activators of NF-κB (*e.g., TLR3* and *TLR4)* and NF-κB target genes that either are (*e.g., CTSB, IL6*) or are not (*e.g., APOE, C3*) SASP components. Transcription of a few genes (*e.g., CXCL3, CXCL5*) rose in the early stages, and then declined. This might suggest a partially successful effort to dampen the inflammatory cycle. Immunoblotting confirmed the presence of transcriptionally active NF-κB from passage 5 to the last passage (Figure 5E). IFN pathways were also upregulated at the mRNA level (Figure 5F). This occurred despite the fact that mRNA FPKM measurement was zero or fractional for IFN-γ, IFN-β, and 13 subtypes of IFN-α, for nearly all samples (Supplementary Table 1), and the highest concentration of secreted IFNs measured was in the low single-digits of picograms per milliliter (Figure 5G). This is less than 0.1% of the amount used to induce transcription of IFN pathway genes in cell culture [31]. Upregulation of IFN signaling pathways despite a lack of IFNs is a phenomenon previously observed in cellular senescence as a consequence of chromatin release into the cytoplasm [15], and in response to chronic mtDNA-mediated cGAS-STING signaling [32]. It likely denotes either a cell-intrinsic, IFN-independent mechanism or an adaptation to chronic, low-level IFN signaling. Among the genes upregulated were a promoter of apoptosis (*XAF1*), and a number of IFN-induced genes (*e.g., CXCL10, IFI6, IFIT1, IFIT2, IFIT3, ISG15, OAS2*). A few IFN pathway genes (*e.g., EGR1, GBP2*, *NUP210*) were downregulated (Figure 5F). Downregulation of *EGR1*, a transcription factor for a suppressor of cytokine signaling, is consistent with upregulation of IFN-like signaling. One of the four p38MAPK genes, *MAPK11*, was significantly increased, and an inhibitor, *CHP2*, was significantly decreased (Figure 5H). The pattern of dominance in upregulation of inflammatory signaling that is seen with the NF-κB and IFN pathways is, however, absent.

One subset of IFN pathway genes, the IFN-related DNA damage resistance signature (IRDS) genes, is noteworthy. The group of 49 IRDS genes has been identified as a “survival signature” found in cancer cells that are resistant to death induced by DNA-damaging therapies [33]. The IRDS genes also substantially overlap a group of genes, induced in response to mtDNA released into the cytoplasm, that promote nuclear DNA repair capacity [32]. The mRNA for 24 of these IRDS genes was differentially expressed in NOKs with senescence, with a strong trend of upregulation (Figure 5I). This suggests that these genes could be productively targeted by senolytic therapies that could also be used in cancer treatments.

The NLRP3 inflammasome, when assembled, activates caspase 1, which cleaves pro-IL-1β and pro-IL-18, after which they are released from the cell [34]. Transcription of genes for IL-1β and NLRP3 (both NF-κB targets) significantly increased with senescence (Supplementary Table 1), the amount of IL-1β in CM increased with senescence (Figure 5C), and the amount of IL-18 (another NF-κB target) in the EV pellet increased with senescence (Supplementary Table 5). We found the NLRP3 inflammasome adaptor protein ASC (PYCARD), in the EV pellet, increasing with senescence (Supplementary Table 5). Thus, senescing NOKs may form inflammasomes that induce secretion of the inflammatory cytokine IL-18 in EVs [35]. In this regard, we found significant (p < 0.05) increases in amyloid-β precursor protein in our proteomic analysis of both CM and the EV pellet (Supplementary Tables 4, 5); amyloid-β is known to activate the NLRP3 inflammasome [36]. Formation of inflammasomes during senescence adds another layer of inflammation to the NOK SASP.

### NOK senescence displays important distinguishing characteristics

**Figure 6:**
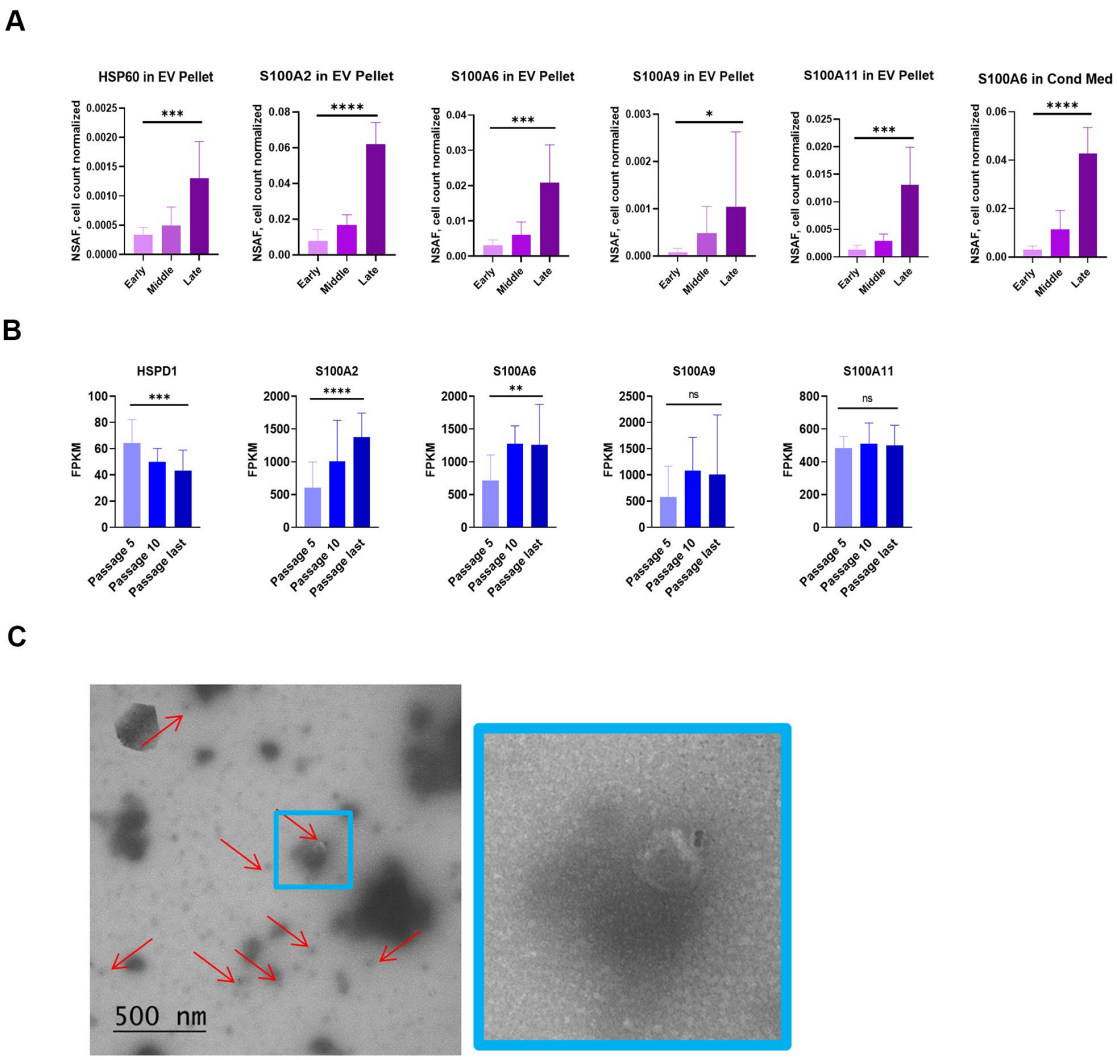
**NOK senescence displays important distinguishing characteristics.** (**A**) Mass spectrometry analysis of proposed SASP additions. Selected proteins significantly increased in EV pellet or CM (Mean ± SD). Y-axis scales differ. (**B**) mRNA levels of proposed SASP additions. (Mean ± SD). Y-axis scales differ. (**C**) TEM of vesicles from EV pellet labelling for surface HSP60 (left, and 4x enlargement, right). For Figure 6A, 6B, *p<0.05, **p<0.01, ***p<0.001, **** p<0.0001.

An important motivation to our work was to identify unacknowledged aspects of senescence that are evidenced more strongly in epithelial cells relative to fibroblasts, or that may be unique to epithelial cells. Based on our mass spectrometry results, we suggest addition of the following proteins to a more epithelial-inclusive SASP: heat shock protein 60 (HSP60), S100A2, S100A6, S100A9, and S100A11. We selected these for two reasons. First, we found these proteins, in quantities that were significantly higher at late passages than early, in the EV pellet or both the EV pellet and CM (Figure 6A). Second, each of these proteins is an endogenous ligand for one of two pro-inflammatory pattern-recognition receptors: the receptor for advanced glycation end products (RAGE) and Toll-like receptor 4 (TLR4) [37–39]. At the mRNA level, only S100A2 and S100A6 significantly increased, and HSP60 mRNA declined significantly with senescence (Figure 6B).

HSP60, one of these nominated proteins, primarily localizes to mitochondria. It has, however, been found in association with the outer surface of the plasma membrane of certain cancer cells and cardiomyocytes, and the membrane of EVs secreted by these cells [38, 39]. We consistently observed, using IF, EVs staining positive for HSP60 outside the NOK cell boundary (Supplementary Figure 6A). To determine whether HSP60 was on the surface of these EVs, we utilized transmission electron microscopy (TEM) and immunogold labeling of EVs enriched from NOK CM. TEM showed EVs that labeled positive for surface HSP60 (Figure 6C, Supplementary Figure 6B), indicating that EV-associated HSP60 is available to interact with receptors on target cells.

### EVs from senescent NOKs activate STING-dependent IFN pathway signaling in THP-1 monocytes

**Figure 7:**
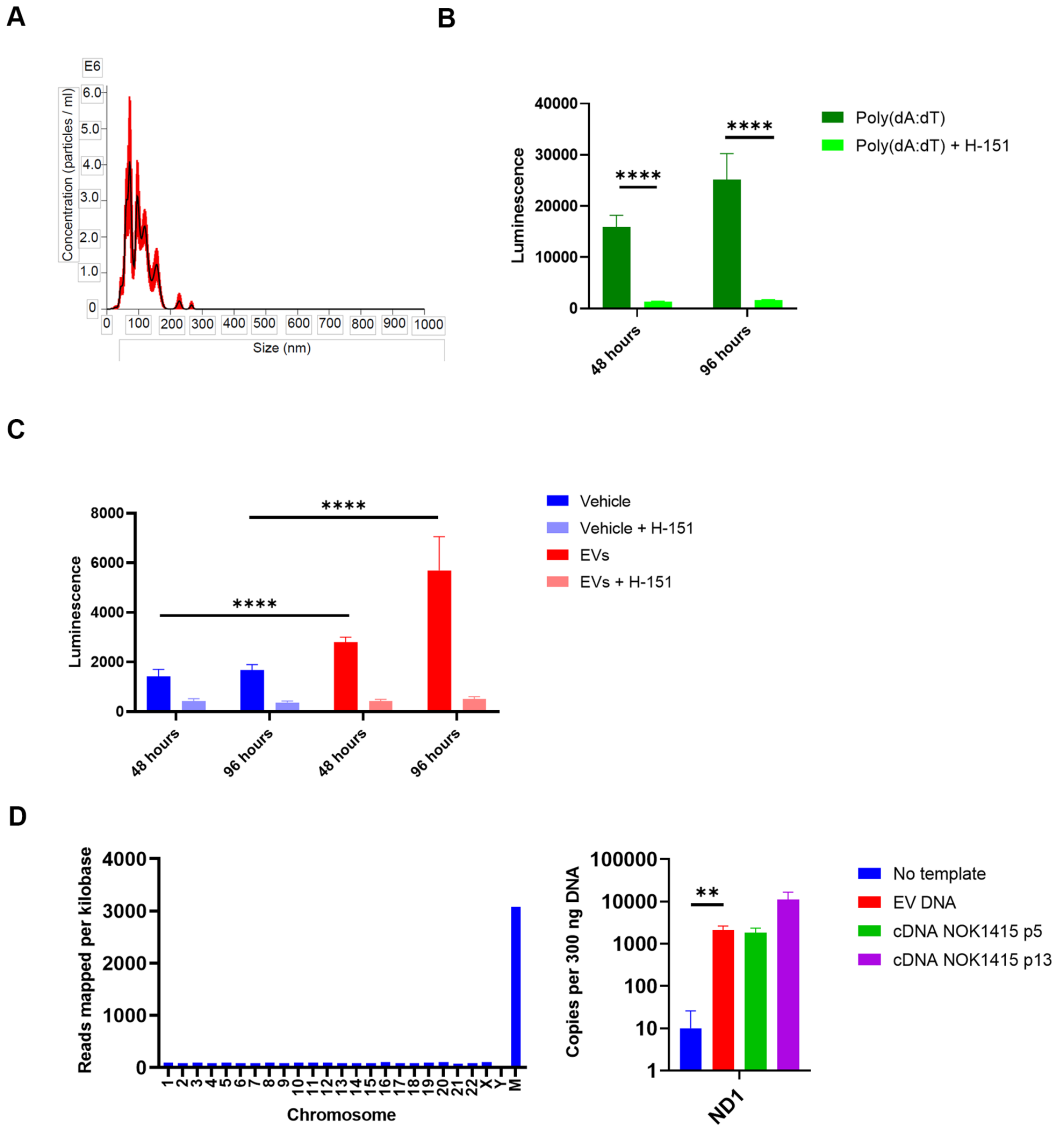
**EVs from senescent NOKs activate IFN pathway signaling in THP-1 monocytes.** (**A**) NanoSight analysis of EVs isolated by SEC shows vesicle size and quantity. (Error bars indicate +/-1 standard error of the mean.) (**B**) IFN transcription in THP-1 cells treated with poly (dA:dT). Over 96 hours, level was strongly reduced by the addition of the STING inhibitor H-151. (**C**) IFN transcription in THP-1 cells treated with EVs. Over 96 hours, level was significantly higher for cells co-incubated with EVs from senescent NOKs compared to vehicle-treated cells, and was significantly reduced by the addition of the STING inhibitor H-151. Results for Figure 7B, 7C show mean of 8 replicates for each condition ± SD. (**D**) DNA is associated with EVs from senescent NOKs. Left, DNA sequencing. Right, ddPCR results. No template reaction was negative control and cDNA from whole cell RNA was positive control. Readings in Figure 7B, 7C were normalized by subtracting mean luminescence of wells containing only Quanti-Luc reagent. Figure7B–7D significance levels determined using t-test. For Figure 7B–7D, *p<0.05, **p<0.01, ***p<0.001, ****p<0.0001.

Having characterized components of the NOK EV SASP, we wondered what effect EVs isolated from the secretions of senescent NOKs might have on inflammatory pathways in another type of cell. We utilized THP-1 monocytes with an IFN reporter system to address this question. These cells secrete luciferase transcribed from a gene under the control of an ISG54 minimal promoter in conjunction with five ISREs. Using size exclusion chromatography, we isolated EVs from late passage CM that was combined from all 3 donors. We utilized an EV fraction whose size was consistent with exosomes and small microvesicles (Figure 7A). We incubated these EVs with the THP-1 cells, either with or without the STING inhibitor H-151 [40], to address the possible role of DNA-mediated IFN signaling. As controls, we used vehicle-treated THP-1 cells and cells treated with the DNA analog poly(dA:dT) in complex with a cationic lipid transfection reagent to facilitate entry into the cells. Effectiveness of STING inhibition by H-151 was demonstrated by the steep reduction in transcription from the IFN pathway binding sites when H-151 was added to poly(dA:dT)-treated cells (Figure 7B).

THP-1 cells showed an increase in transcription from the IFN reporter when incubated with EVs from senescent NOKs, which was sharply reduced by the addition of H-151. (Figure 7C). Given that cGAS-cGAMP-STING signaling is activated by cytosolic DNA, this suggested that the increase in IFN pathway signaling caused by EVs is mediated in large part by nuclear DNA, mtDNA, or both. Accordingly, we isolated DNA from the same batch of EVs used for the THP-1 experiment, in order to verify its presence. DNA sequencing showed that DNA from all human chromosomes was detected. There was relatively even coverage for the autosomal chromosomes (average of 87 reads per kilobase for chromosomes 1-22), and coverage across mtDNA was > 35-fold enriched in comparison, at 3,079 reads per kilobase (Supplementary Table 6, Figure 7D). We confirmed the presence of mtDNA in EVs using droplet digital PCR, which allows for absolute quantification of target DNA copies (Figure 7D).

These data demonstrate that the vesicular components of the NOK SASP can induce inflammatory signaling in at least one type of immune cell and that part of this inflammation is likely due to DNA carried by the vesicles. It is also possible that interaction with EVs triggers signaling events that lead to cytoplasmic release of nuclear or mitochondrial DNA in the affected cells.

## DISCUSSION

In this study, we carried out a multi-pronged inquiry into senescence in human NOKs, as a representative epithelial cell. We believe the study constitutes a unique combination of transcriptome and secretome senescence profiling, by its use of a population of cells that senesce at different rates. This serves as an accelerated version of organismal cellular senescence, allowing us to create a profile that reflects the interplay of senescent and non-senescent cells. Because carcinomas derive from epithelial cells, the results should also be relevant to age-related cancer risk.

We showed that NOKs display many of the known characteristics of senescence as they are passaged (Figure 1). Among these indicia of senescence, we observed both a significant increase in the number of unresolved double-stand DNA breaks, evidenced by the number of γH2AX foci, and positive staining for γH2AX in the cytoplasm. This is consistent with prior work establishing the existence of cytoplasmic chromatin fragments containing DNA that bleb off from the nucleus in senescence and trigger cGAS-STING signaling [15].

In contrast to fibroblasts, NOKs showed substantial p21WAF1/CIP1 in early passages, which decreased with senescence (Figure 1). Prior NOK studies have not been in accord on changes in p21WAF1/CIP1 in senescing NOKs. One study found that p21WAF1/CIP1 mRNA increased in senescent cells versus highly proliferating cells [12], another found it nearly unchanged [10], and a third found a significant diminution in p21 protein with senescence [13]. We show that p21 mRNA is transcribed in cultures of largely proliferating, mixed, and majority non-proliferating NOKs. p21 protein is also produced at early passages, and declines with senescence. While this means that p21 is not a reliable marker of NOK senescence, it does not allow a conclusion that p21 does not play a role in that process; the multiple functions it performs may change as the cells are passaged. In addition to mediating cell cycle arrest, p21 is active in DNA repair and apoptosis [41].

Our unbiased profile of the senescing NOK transcriptome highlighted both the intensity of the upregulation of inflammatory factors and major changes in cancer-associated genes. The preponderance of genes whose changes are correlated with cancer and the role of senescence in cancer avoidance suggests that other differentially expressed genes we identified may have as-yet unknown connections to cancer. As a caveat, if prior studies showing an association between cancer and transcriptional changes used tumor samples from individuals who had received therapy to induce senescence in their cancer cells, that work might actually be showing correlation between senescence and variations in transcription. Moreover, three of the genes on our top 100 lists (*PAGE3, BGN*, and *GABRQ*) are on the X chromosome and their quantities could be over-represented if dosage compensation were lost in a female donor. Conversely, only one of the three donors was male, and Y chromosome genes, while few in number, could be under-represented in our dataset. We also identified transcription factors likely responsible for upregulation and downregulation of transcription that occur with senescence. This indicated an upregulation of inflammation, particularly IFN pathways. We also identified a separate group of transcription factor binding motifs in genes whose transcription was significantly downregulated in the early stages of senescence. Prominent among the target genes for these transcription factors are genes active in cell cycle and DNA repair.

While a decline in expression of DNA damage repair gens in senescence has been previously seen, our observations disclosed differences between donors for mRNA from many genes involved in the process of DNA damage repair. Further investigation showed that the decrease in E2F1 mRNA with senescence for the female donors, but an increase for the male donor, was accompanied by corresponding changes in protein level. While Rad51 protein increased with senescence for donors of either sex, the increase was much more significant for the male donor, consistent with the upregulation of transcription for the male donor. Sex differences in DNA repair capacity and consequent susceptibility to certain cancers have previously been observed [42]. Thus, the divergent trend may be most relevant when the senescence machinery is unable to enforce replicative arrest of potentially cancerous cells [43]. Based solely on a comparison of three donors, we cannot attribute the differences in transcription and protein for DNA repair genes to sex, or to some other factor specific to a donor. However, the matter bears further investigation and underscores the importance of studying cells and organisms exhibiting diverse traits.

We were also able to compare our RNA-seq results with those of a prior study that defined a core signature of senescence in other cell types, showing that slightly more than half of the genes constituting that signature were also significantly changed in NOKs. The genes conserved between the two data sets are involved in a range of processes, including transcription, apoptosis, cell cycle, and cell growth. Cyclin D1 (*CCND1*), for example, prevents entry into S-phase [44]. Some (*e.g., GDNF*) have been found to be associated with cancer. Of particular interest is that transcription of the *BCL2L2* gene was increased with senescence in the core signature, but decreased in NOKs. This implies that a senolytic treatment that would be effective in other cells by virtue of inhibiting Bcl-2-like protein 2 would not induce apoptosis in senescent NOKs. Transcription of another anti-apoptotic gene, *BCL2A1*, however, was significantly upregulated in NOKs (Supplementary Table 1).

We conducted an unbiased analysis of the senescing NOK secretome, examining both CM and EVs, identifying the top proteins that increased or decreased with senescence and the roles they play. While some of these proteins can be associated with vesicles or not, differences between proteins secreted in the soluble fraction and those secreted in vesicles suggest that senescing cells utilize distinct mechanisms to expel certain proteins. This is likely to affect the ability of these proteins to interact with other components of plasma and the probability that they will be taken up by other cells. Although most of the secretome proteins are not components of the SASP as it is currently defined, several are nevertheless capable of producing chronic inflammation. Prominent among these proteins were DAMPS. Focusing on the early stages of senescence, our analysis identified a strong decline in a particular ribosomal protein. However, the EV pellets obtained via ultracentrifugation, long considered the gold standard for EV isolation, do contain some non-vesicular protein aggregates (*e.g.*, major vault protein).

Our RNA seq data showed that while many SASP factors were upregulated at the transcriptional level, not all cell types senesce in an identical fashion. We verified secretion of a subgroup of soluble SASP factors. A number of them are associated with age-related frailty and disease. The cytokine IL-6, the protease inhibitor PAI-1 (SERPINE1), and the protease urokinase plasminogen activator (uPA) have been proposed as biomarkers of age-associated frailty due to their prevalence and effects in the pathologies of aging [45]. The protease MMP-1 has also been identified as an aging biomarker [25].

We placed the upregulation of inflammation with NOK senescence in the context of the SASP and known signaling pathways: NF-κB, IFN, and p38MAPK. We found that while NF-κB and IFN pathways were strongly upregulated, mRNA increased much more selectively for elements of the p38MAPK inflammatory pathways. By looking at which components of those three pathways are significantly changed, we have highlighted elements that may be specific to NOK or epithelial cell senescence. In particular, we identified a group of IFN pathway genes upregulated with senescence that overlaps with the IRDS signature in cancers that are resistant to radiation and chemotherapy.

Based on our proteomic findings, we also proposed five (HSP60 and four S100A proteins) additions to the SASP, each of which can bind RAGE or TLR4. Not only are these receptors found on immune cells, but TLR4 was significantly upregulated at the mRNA level as NOKs senesce (Supplementary Table 1), suggesting a mode for transmitting or reinforcing senescence in a paracrine manner between NOKs by activation of NFκB. One of the additions we nominate, HSP60, is appended to the membrane of EVs released by senescing NOKs. Such an event by senescing cells has been the subject of some speculation [46], but has not previously been shown.

The field of senescence research has, in recent years, been moving toward an appreciation of the importance of EVs [25]. EV content, by virtue of its surrounding membrane, has a passport to destinations that many naked proteins and nucleic acids cannot reach. Our experiment with THP-1 reporter cells showed that senescent NOK EVs can activate IFN pathways in monocytes. The length of time that the activation persists may be a consequence of the period over which EVs can be taken up by the THP-1 cells. We found that these EVs are associated with DNA, which can trigger cGAS-STING signaling. Although we did not reach a conclusion about whether the DNA was internal to the EV, or externally appended, DNA can be taken up into the cytoplasm of a target cell in either case, as EVs can be internalized by several possible interactions [14]. Vesicular secretion of large amounts of extracellular mtDNA has been found in studies of cancer cell lines [47], but less often in senescence research [48]. It has been suggested that the generation of vesicles derived from mitochondria is instigated by oxidative damage [49]. As yet unknown is whether specific portions of the mitochondrial genome are more likely to be released from the mitochondria in association with vesicles. We anticipate that continued active investigation of the generation, content, and impact of EVs will yield further insights into how to ameliorate senescence.

Simultaneous application of multiple profiling methods to other types of cells will disclose how widely the behavior of senescent NOKs is shared by other cells. Robust profiling can be used to address important remaining questions, such as explication of the mechanisms of inflammation and immune evasion by senescent cells. Critically, we also must assess which aspects of *in vitro* senescent cells are most prevalent *in vivo* among individuals suffering from the pathologies of aging. A comparison of EVs from specific types of senescent cells to those found in these individuals can help determine which EVs, or their source cells, could be therapeutically targeted. A recent proteomic analysis of Alzheimer’s disease (AD) brain and cerebrospinal fluid (CSF) identified astrocyte/microglial metabolism proteins that were significantly increased in AD CSF of a cohort of patients compared to a control group [50]. Five of the most significantly increased were also significantly up in the NOK EV pellet, comparing late to early passages: CD44, peroxiredoxin 1, lactate dehydrogenase B-chain, pyruvate kinase, and GAPDH. This overlap illustrates how basic profiling of senescence in multiple cell types, and specifically in normal cells with an intact capacity to undergo senescence spontaneously, can help us develop targeted therapies.

## MATERIALS AND METHODS

### Cell culture

NOKs were collected at the National Institutes of Health during third molar extractions and isolated according to published procedures [51]. NOKs were cultured on surfaces coated with collagen I protein (Gibco #A1048301) in Defined Keratinocyte Serum-Free Medium (DKSFM) (Life Technologies #10744019) with penicillin-streptomycin (Gibco #15140163). 5x10^5^ cells were plated in a 10 cm dish in 10 mL of DKSFM and passaged every 4 days through passage 11 and every 5 days thereafter to determine growth rates. Medium was replaced at 48 hours after passaging and was replaced or cells were passaged at 96 hours. Collected medium was centrifuged for 5 minutes at 1500 x g. Supernatant was transferred to fresh tubes and stored at -80° C. All cells used tested negative for mycoplasma using the MycoAlert Mycoplasma Detection Kit (Lonza #LT07-318).

### RNA-seq

10^5^ NOKs were plated in each of 2 wells of a 6-well plate and grown for 5 days. Cells were collected in TRIzol (Life Technologies #15596026) and stored at -80° C. RNA was extracted using the Direct-zol RNA Miniprep kit (Zymo Research #R2050). Quality of isolated total RNA was assessed using Agilent TapeStation 4200. RNA-Seq libraries were prepared using the TruSeq Total mRNA Sample Preparation Kit (Illumina). RNA-seq libraries were multiplexed, normalized, pooled for sequencing, and sequenced on the HiSeq 2500 system (Illumina) at single read 50. Image analysis and base calling was done with Illumina CASAVA-1.8.2.

### RT-qPCR

cDNA was prepared from 1 μg of RNA (same samples used for RNA-seq) in a 20 μl reaction using qScript cDNA SuperMix (Quanta #84034). cDNA was diluted 1:10 in TE buffer. qPCR was performed with 2.5 μl cDNA in a 20μl reaction using Power SYBR Green PCR Master Mix (Applied Biosystems #436789) and primers specified in Supplementary Table 7. Each sample was run in triplicate on a BioRad CFX384 Real Time machine as follows: 50° C for 2 minutes, 95° C for 10 minutes, and 40 cycles of 95° C for 15 seconds followed by 60° C for 1 minute.

### Senescence associated β-galactosidase

NOKs were stained for SA β-gal (BioVision #K320-250) 4 days after passaging. Etoposide-treated (20 μM for 48 hours) BJ fibroblasts were the positive control. Cells were imaged 40 hours after application of stain. At least 500 cells were counted at each passage for each of four replicates.

### EdU and FxCycle violet

Cells were stained using the Click-iT EdU Flow Cytometry Assay Kit (Invitrogen #C10424), and FxCycle Violet Stain (Invitrogen #F10347). Treated cells were incubated with 10 μM EdU for 24 hours. U2OS cells were the positive control. NOKs not treated with EdU were the negative control. Cells were analyzed on a Becton-Dickinson LSRII flow cytometer. Results were plotted using FlowJo v10 software (FlowJo LLC).

### γH2AX

NOKs were stained with an AF488-conjugated antibody (Cell Signaling Technology #9719) at a 1:1500 dilution and DAPI (ThermoFisher #D1306) per a published protocol [52]. Etoposide-treated (2 μM for 48 hours) U2OS cells were the positive control. Images were acquired with a Zeiss 880 confocal (63x objective, zoom = 2). Images were randomized to perform manual scoring blind to passage number. At least 200 cells from each of passages 5 and 13 were scored.

### Immunofluorescence for cyclin-dependent kinase inhibitors, HSP60, and Rad51

NOKs were cultured in 4-well collagen-coated glass slides (Millicell PEZGS0416). Cells were fixed with 4% paraformaldehyde and permeabilized with 0.2% Triton/3% FBS in PBS. Antibodies to p16INK4a (R&D Systems #AF5779), p21CIP1/WAF1 (ThermoFisher #MA5-14949), HSP60 (Cell Signaling Technology #12165S), and Rad51 (Abcam #63801) and secondary antibodies conjugated to AF647 (Invitrogen #A-21447) for p16 and AF488 (Invitrogen #A-21202 and #A-21206) for p21, HSP60, and Rad51 were used, as well as DAPI (ThermoFisher #D1306). Phalloidin conjugated to AF568 (ThermoFisher #A12379) was used to stain for actin. Slides were mounted using ProLong Glass Antifade Mountant (Invitrogen #P36980) and Slip-Rite Cover Glass (Thermo Scientific #152250). Positive staining for p21 at an early passage was confirmed using a second primary antibody (Cell Signaling Technology #2947). Etoposide-treated (2 μM for 48 hours) U2OS cells were the positive control for p21 and Rad51, and etoposide-treated (20μM for 48 hours) BJ fibroblasts were the positive control for p16. p16INK4a and Rad51 fluorescence were quantified using ImageJ.

### Immunoblotting

Cells were washed with PBS and collected in RIPA buffer with protease/phosphatase inhibitor (Cell Signaling Technologies #55872S). Protein quantification was performed using Bio-Rad DC Protein Assay reagents (Bio-Rad # 5000113, #5000114, and #5001155); readings were made on an Infinite M200 Pro plate reader (TECAN) at 750 nm. Protein content was normalized to the lowest concentration sample using the same buffer. Samples were prepared for immunoblotting with NuPAGE Sample Reducing Agent (Invitrogen #169323) and NuPAGE LDS Sample Buffer (Invitrogen #1771559), heated for 15 minutes at 70° C. Samples were run on NuPAGE 4-12% Bis-Tris gels (Invitrogen #NP0322BOX) using MOPS running buffer (Invitrogen #NP000102). Transfers were run using NuPAGE transfer buffer (Invitrogen #NP00061) onto nitrocellulose membranes (Thomas Scientific #1182G93). After transfer, Ponceau S staining was performed using 10x Ponceau S solution (0.5% (w/v) Ponceau S powder (Sigma-Aldrich #P3504) dissolved in 1% (v/v) glacial acetic acid) that had been diluted to 1x with sterile H20. Ponceau S stain was removed by incubating the stained membrane in 0.1N NaOH followed by washing in H2O. Membranes were blocked in TBST with 5% milk (American Bio AB10109-01000) and probed using antibodies to p21WAF1/CIP1 (Cell Signaling Technology #2947S), E2F1 (Santa Cruz Technology #SC-251), phosphorylated p-65 (S536) (Cell Signaling Technology #3033S), or β-actin conjugated to HRP (Cell Signaling Technology #5125S), overnight at 4° C in blocking buffer. For primary antibodies not conjugated to HRP, anti-rabbit or anti-mouse secondary antibodies so conjugated were used (Bio-Rad #1706515 and #170516) for one hour at room temperature in blocking buffer. Membranes were treated with Lumina Forte HRP Substrate (Millipore Sigma # WBLUF0100) and imaged on a ChemiDoc MP imaging system (BioRad). Bands were quantified using ImageJ.

### Cytokine analysis

Medium was collected at 96 hours after passaging. Analysis was performed by Eve Technologies (Array #HD42) using a multiplex immunoassay kit (Millipore Sigma #HCYTMAG60PMX41BK) and analyzed with a Bio-Plex 200 system (Bio-Rad). Results were normalized for cell number and the amount of each protein present in clean DKSFM.

### EV isolation for proteomics

Frozen CM was thawed on ice, centrifuged at 300 x g for 5 minutes at 4° C and then 2,000 x g for 10 minutes at 4° C. The supernatant was transferred to fresh tubes and centrifuged at 10,000 x g for 30 minutes at 4° C. 500 μL of medium was taken as the CM sample. The remainder was filtered through a 200 nm pore nylon filter (Pall Corp. #PN4433) and centrifuged in an Optima L-80XP using an SW-32 Ti rotor (Beckman Coulter) at 100,000 x g for 70 minutes at 4° C; the pellet was washed with ice-cold PBS, and centrifuged at 100,000 x g for 70 minutes at 4° C. Supernatant was removed and the pellet and CM sample were stored at -80° C.

### Mass spectrometry

Samples were precipitated by methanol/chloroform and redissolved in 8 M urea/100 mM TEAB, pH 8.5. Proteins were reduced with 5 mM tris(2-carboxyethyl)phosphine hydrochloride (TCEP) (Sigma-Aldrich) and alkylated with 10 mM chloroacetamide (Sigma-Aldrich). Proteins were digested overnight at 37° C in 2 M urea/100 mM TEAB, pH 8.5, with trypsin (Promega). Digestion was quenched with formic acid, 5% final concentration. The digest was injected directly onto a 30 cm, 75 μm ID column packed with BEH 1.7um C18 resin (Waters). Samples were separated at a flow rate of 200 nl/min on a nLC 1000 (Thermo). Buffer A and B were 0.1% formic acid in water and 0.1% formic acid in 90% acetonitrile, respectively. A gradient of 1-25% Buffer B over 110 minutes, an increase to 40% Buffer B over 10 minutes, an increase to 90% Buffer B over 10 minutes and held at 90% Buffer B for a final 10 minutes was used for 140 minutes total run time. Column was re-equilibrated with 15 μl of Buffer A prior to the injection of sample. Peptides were eluted directly from the tip of the column and nanosprayed directly into the mass spectrometer by application of 2.5 kV voltage at the back of the column. Samples were analyzed on a Fusion Orbitrap tribrid mass spectrometer (Thermo). The Orbitrap Fusion was operated in a data dependent mode. Full MS scans were collected in the Orbitrap at 120K resolution with a mass range of 400 to 1500 m/z and an AGC target of 4e5. The cycle time was set to 3 seconds, and within the 3 seconds the most abundant ions per scan were selected for CID MS/MS in the ion trap with an AGC target of 1e4 and minimum intensity of 5000. Maximum fill times were set to 50 ms and 100 ms for MS and MS/MS scans respectively. Quadrupole isolation at 1.6 m/z was used, monoisotopic precursor selection was enabled and dynamic exclusion was used with exclusion duration of 5 sec. Protein and peptide identification were done with Integrated Proteomics Pipeline – IP2 (Integrated Proteomics Applications). Tandem mass spectra were extracted from raw files using RawConverter and searched with ProLuCID against Uniprot human database. The search space included all fully-tryptic and half-tryptic peptide candidates. Carbamidomethylation on cysteine was considered as a static modification. Data was searched with 50 ppm precursor ion tolerance and 600 ppm fragment ion tolerance. Identified proteins were filtered using DTASelect and utilizing a target-decoy database search strategy to control the false discovery rate to 1% at the protein level. Normalized spectral abundance factor (NSAF) was calculated as the number of spectral counts (SpC) identifying a protein divided by the protein’s length (L), divided by the sum of SpC/L for all proteins in the experiment.

### Transmission electron microscopy

Exosomes isolated by ultracentrifugation were prepared for TEM using a published protocol [53]. Fresh exosomes were negatively stained using 1% aqueous uranyl acetate for 1 minute. Exosomes fixed with 2% PFA were transferred to carbon-coated nickel grids, incubated with primary antibodies to CD9 (ThermoFisher #10626D), CD81 (ThermoFisher #10630D), or HSP60 (Cell Signaling Technology #12165S) at a concentration of 10 μg/μL. Secondary antibodies conjugated to 10 nm gold beads were donkey anti-mouse IgG (Electron Microscopy Sciences #25813) for CD9 and CD81 and goat anti-rabbit IgG (Electron Microscopy Sciences #25365) for HSP60. Images were acquired using a Leo Libra 120kV Transmission Electron Microscope (Carl Zeiss) equipped with a Gatan 4k 895-Ultrascan CCD camera, operated at 80 kV using Zero-loss imaging to increase contrast.

### THP-1 cell co-incubation

Frozen CM from the final or penultimate passages from each donor was thawed on ice, centrifuged at 300 x g for 5 minutes at 4° C and 2,000 x g for 10 minutes at 4° C. Supernatant was transferred to fresh tubes and centrifuged at 10,000 x g for 30 minutes at 4° C. Supernatant from this last centrifugation step was filtered through a 200 nm pore nylon filter (Pall Corp. #PN4433). EVs were isolated using the qEV10/35nm size exclusion column (Izon). Fractions were analyzed using a NanoSight NS300 with NTA 3.1 software (Malvern Panalytical), then concentrated 8:1 by centrifuging at 3220 x g in Amicon® Ultra-15 3K filters (Millipore #UFC900308). Protein content was determined as for immunoblot samples. THP-1 Dual Reporter cells (InvivoGen #thpd-nfis) were cultured in RPMI1640 (ThermoFisher 11875093) with Glutamax (Gibco 35050-061), HEPES (Gibco # 15630130), 10% FBS (Gibco 10437-028) heat-inactivated at 56° C for 30 minutes, 100 μg/ml Normocin (InvivoGen #ant-nr-1), and penicillin-streptomycin (Gibco #15140163). Blasticidin (InvivoGen #ant-bl-1) at 10 μg/ml and Zeocin (InvivoGen #ant-zn-1) at 100 μg/ml were added to the medium at alternate passages for selection. THP-1 cells were plated (1.5 x 10^5^/well in a 24-well plate) in 500 μl medium. 5 μg of EVs in sterile PBS were added to treated wells; an equal amount of sterile PBS was added to negative control wells. For the positive control, poly (dA:dT) complexed with LyoVec (InvivoGen #tlrl-piclv) was added to a final concentration of 100 ng/ml. H-161 (Invivogen #inh-h151) was used at a concentration of 500 ng/mL added every 24 hours, starting one hour before addition of EVs. Secretion of luciferase was detected using Quanti-Luc (InvivoGen #rep-qlc). Readings were performed using the luminescence function of the Infinite M200 Pro plate reader (TECAN) with a read time of 100 milliseconds.

### DNA Extraction and sequencing

DNA was extracted from EVs using XCF Exosomal DNA Isolation Kit (System Biosciences #XCF200A-1) according to manufacturer’s protocol. Library preparation was performed using Nextera XT DNA kit (Illumina #FC-131-1096) according to the manufacturer's instructions and DNA libraries were sequenced on NextSeq500 System (Illumina) using the mid-output kit at paired-end 75bp configuration.

### Droplet digital PCR (ddPCR)

ddPCR was carried out using the QX-200 system (Bio-Rad) according to the manufacturer’s user guides. Samples were prepared using 11 μL of ddPCR Supermix for Probes (no UTP) (Bio-Rad #1863023), 1.1 μL of 20x target primers/probe (Bio-Rad # 10031276), 330 ng DNA, and UltraPure DNase/RNase-Free Distilled Water (Gibco # 10977015) to a total volume of 22 μL, of which 20 μL was used for each reaction. A reaction with no DNA template was used as negative control and cDNA made from RNA from passage 5 and last passage whole cells was used as positive control. Primer and probe sequences specified in Supplementary Table 7.

### Bioinformatics

For RNA-seq, sequenced reads were quality-tested using FASTQC (https://www.bioinformatics.babraham.ac.uk/projects/fastqc/) v0.11.8 and aligned to the hg19 human genome using the STAR aligner version 2.5.3a. Mapping was carried out using default parameters, filtering non-canonical introns and allowing up to 10 mismatches per read and only keeping uniquely mapped reads. The genome index was constructed using the gene annotation supplied with the hg19 Illumina iGenomes collection (iGenomes online. Illumina. 2015. http://support.illumina.com/sequencing/sequencing_software/igenome.html) and sjdbOverhang value of 100. Raw or FPKM (fragments per kilobase per million mapped reads) gene expression was quantified across all gene exons with HOMER v4.10.4 analyzeRepeats.pl with hg19 annotation v6.4 and parameters -strand + -count exons -condenseGenes (top-expressed isoform as proxy for gene expression), and differential gene expression was carried out on the raw counts with HOMER getDiffExpression.pl that runs DESeq2 v1.14.1 using replicates to compute within-group dispersion and experimental batch modeled as a separate variable. Principal Component Analysis (PCA) was carried out on normalized filtered gene counts using the R prcomp function.

Motif enrichment analysis and motif searching was carried out with default HOMER findMotifs.pl with 1000 replicates for FDR calculations.

Overrepresentation analysis was carried out with WebGestalt, using the GO biological process non-redundant database with FDR < 0.01 as the significance threshold, protein coding genes as the reference list, a maximum number of genes in a category of 200, using a weighted set cover algorithm to minimize the number of significant terms to 10.

Differential proteomics analysis was carried out using trended robust empirical Bayes testing from the limma R package version 3.38.3 on preprocessed protein counts (NSAF corrected for cell number, log2 transformed, and filtered for mean expression > 0.0001, resulting in 915 proteins tested) and accounting for batch effects between experiment batches.

For DNA sequencing, samples were mapped to the hg38 human genome using STAR v2.5.3a a and the non-repeat sequences were used to quantify the length-normalized coverage of reads across each chromosome. Since samples represent technical replicates, the aligned reads were merged with samtools v1.9. The total number of reads aligning to each chromosome, and the repeat-masked genome length (with repeat sequences removed), were used to calculate the normalized read count for each chromosome.

### Data availability

The data that support these findings are openly available as Supporting Information. RNA-seq results have been posted to the Gene Expression Omnibus (GEO) database (https://www.ncbi.nlm.nih.gov/geo/query/acc.cgi?acc=GSE155371).

## Supplementary Material

Supplementary Figures

Supplementary Table 1

Supplementary Table 2

Supplementary Table 3

Supplementary Table 4

Supplementary Table 5

Supplementary Table 6

Supplementary Table 7
