# Supplementary material for: Insights into epithelial cell senescence from transcriptome and secretome analysis of human oral keratinocytes
Source: Aging (Albany NY). 2021 Feb 12;13(4):4747–77. doi: 10.18632/aging.202658 (PMC7950289; doi:10.18632/aging.202658)
Supplement: Supplementary Figures [file aging-13-202658-s001.pdf]

SUPPLEMENTARY FIGURES

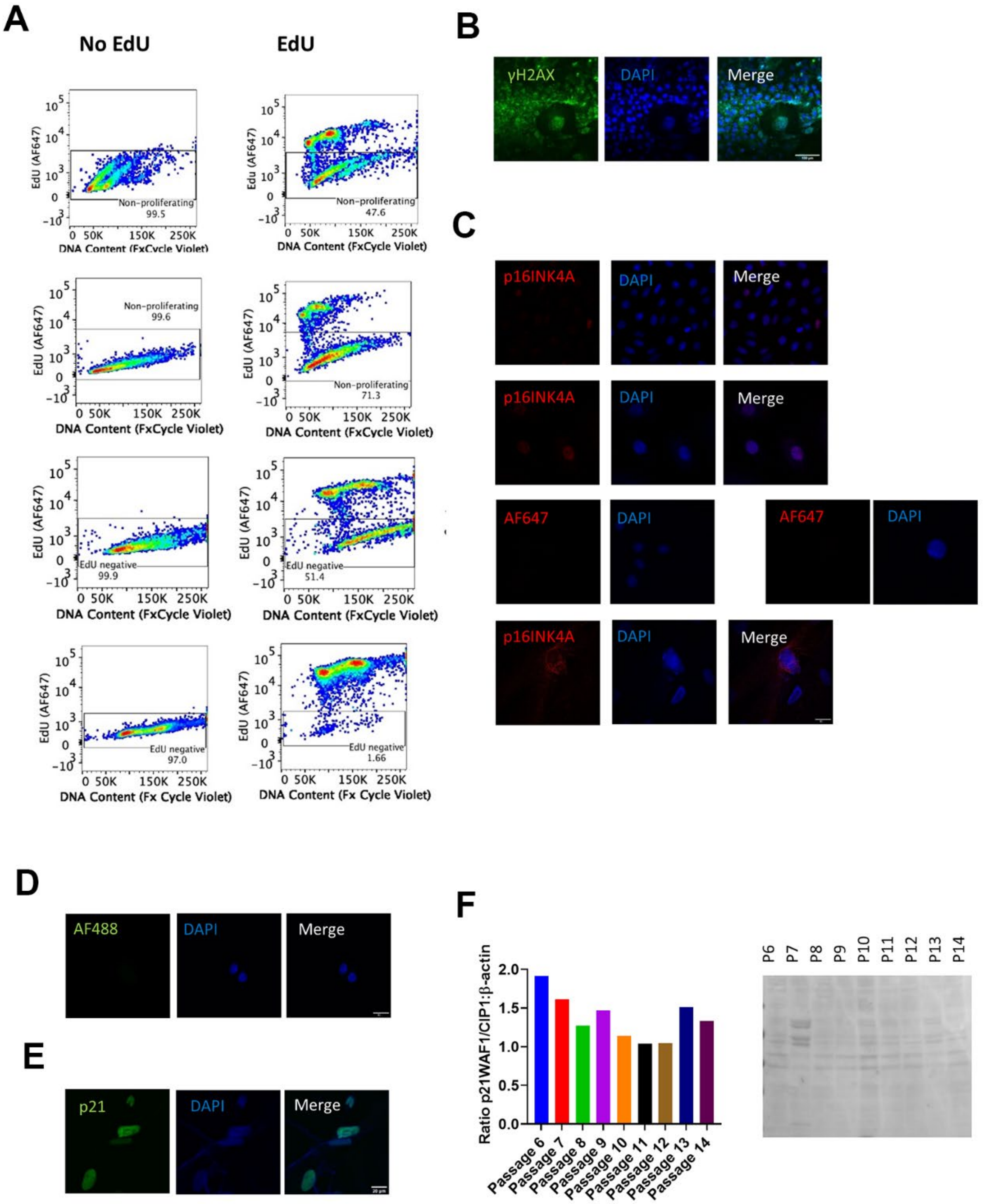

Supplementary Figure 1. NOK senescence assays. (**A**) Flow cytometry for additional NOK donors, including negative controls (no Edu treatment) and U2OS positive control. (Top two rows, Donor 1408, passage 10 and last passage; third row, Donor 1508 passage 10; fourth row, U2OS cells). (**B**) IF for γH2AX positive control U2OS cells. (**C**) IF for p16INK4A. (Top, passage 5; second row, passage 13). Fluorescence per cell for p16INK4A protein as passage 13 is 2.87 times the level at passage 5 ($p<0.0001$ using two-tailed t-test) (Donor 1415) (data not shown). If for p16INK4A negative control NOKs (no primary antibody) (third row left, passage 5; third row right, passage 13) (Donor 1415). If for positive control etoposide-treated BJ fibroblasts (fourth row). (**D**) IF for p21WAF1/CIP1 negative control NOKs (no primary antibody) (Donor 1408, passage 5). (**E**) If for p21WAF1/CIP1 positive control U2OS cells. (**F**) p21WAF1/CIP1 ImageJ quantification reading normalized to β-actin (left). Similar results were obtained for normalization to total protein in Ponceau S Stain (right).

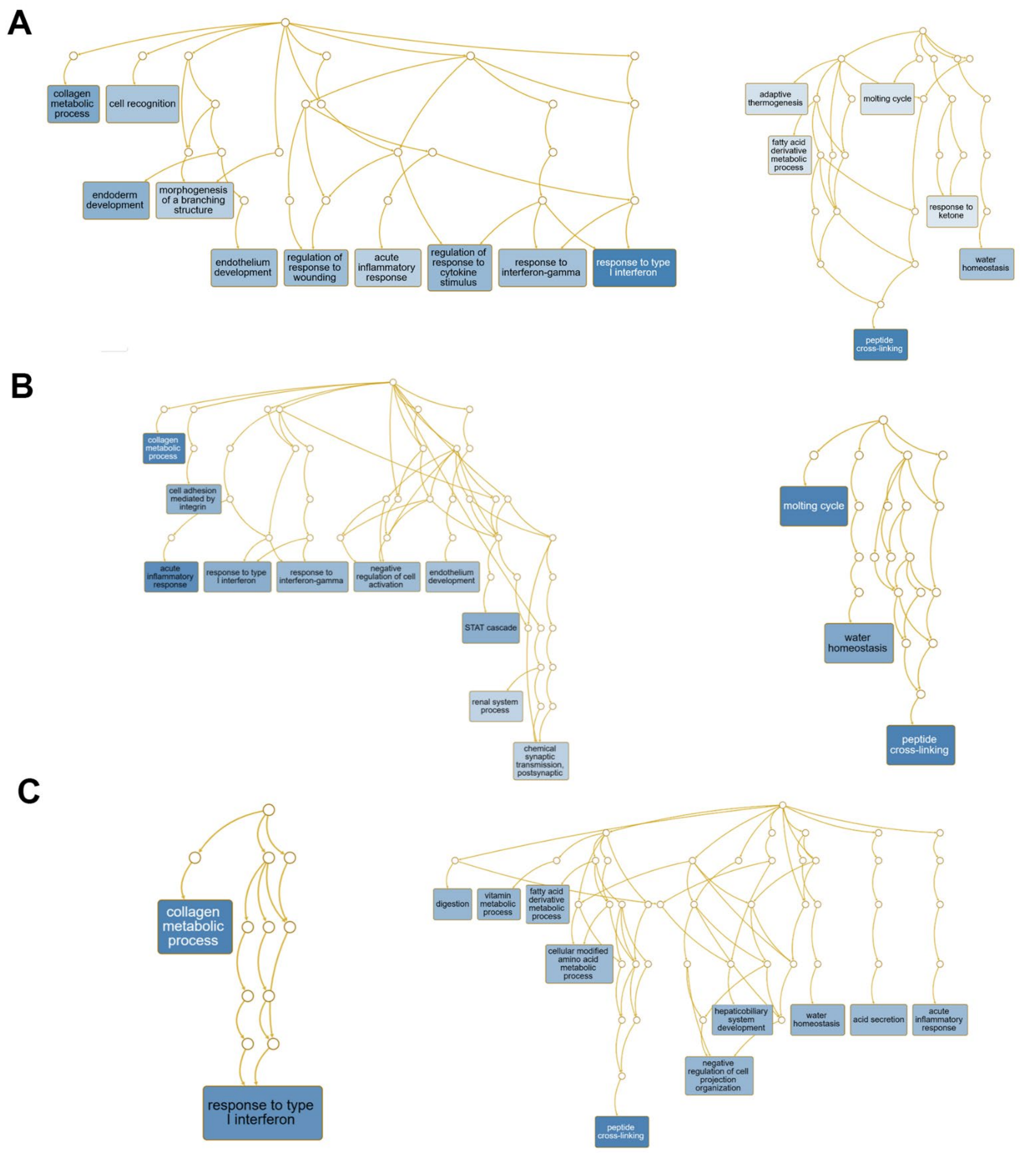

Supplementary Figure 2. Overrepresentation analysis. (**A**) WebGestalt directed acyclic graph (DAG) (using weighted set cover) for genes upregulated with senescence shows upregulation of inflammatory processes from passage 5 to last passage (left). DAG for genes downregulated with senescence shows downregulation of peptide cross-linking from passage 5 to last passage (right). (**B**) DAG for genes upregulated with senescence shows upregulation of inflammatory processes from passage 5 to passage 10 (left). DAG for genes downregulated with senescence shows downregulation of peptide cross-linking from passage 5 to passage 10 (right). (**C**) DAG for genes upregulated with senescence shows upregulation of response to type I interferons from passage 10 to last passage (left). DAG for genes downregulated with senescence shows downregulation of acute inflammatory response from passage 10 to last passage (right), which represents a different set of genes, with one exception, than those upregulated comparing passage 5 to passage 10.

A

	Motifs Enriched in Promoters of Upregulated Genes (Passage last vs passage 5)	q-value/FDR	% of Targets	% of Background	Best Match/Details
1	CAGGGAATTTCC	0.001	1.35%	0.05%	NFkB-p65-Rel(RHD)/ThioMac-LPS-Expression(GSE23622)/Homer(0.917)
2	AGGTTACTCA	0.001	6.67%	2.30%	Fra1(bZIP)/BT549-Fra1-ChIP-Seq(GSE46166)/Homer(0.733)
3	GAAACCGAAACT	0.003	2.98%	0.57%	ISRE(IRF)/ThioMac-LPS-Expression(GSE23622)/Homer(0.941)

B

	Motifs Enriched in in Promoters of Downregulated Genes (Passage last vs passage 5)	q-value/FDR	% of Targets	% of Background	Best Match/Details
1	CATGTCTGGTCG	0.015	0.77%	0.00%	PB0060.1_Smad3_1/Jaspar(0.738)
2	ATTTTCGCGCCC	0.043	2.13%	0.28%	E2F7(E2F)/Hela-E2F7-ChIP-Seq(GSE32673)/Homer(0.800)
3	AGTCTATAAAGG	0.043	2.13%	0.29%	TATA-Box(TBP)/Promoter/Homer(0.621)
4	TATAAAGA	0.003	9.86%	4.75%	POL012.1_TATA-Box/Jaspar(0.896)
10	GTGTTTAT	0.004	13.01%	7.31%	FOXL1/MA0033.2/Jaspar(0.846)

C

#	Motifs Enriched in Promoters of Downregulated Genes (Passage 10 vs Passage 5)	q-value	% of Targets	% of Backgrounds	Best Match
1	ACGCAAATAAGG	0.002	3.83%	0.10%	Gata1(Zf)/K562-GATA1-ChIP-Seq(GSE18829)/Homer(0.655)
2	GTATATAAAAGC	0.007	5.43%	0.39%	TBP/MA0108.2/Jaspar(0.725)
3	GGGCAAGTGACC	0.016	2.56%	0.02%	USF1/MA0093.2/Jaspar(0.757)
4	TGTGCCCTGTGG	0.025	5.43%	0.49%	AP-2alpha(AP2)/Hela-AP2alpha-ChIP-Seq(GSE31477)/Homer(0.748)
5	GAATTGTAAATA	0.025	3.51%	0.12%	PB0165.1_Sox11_2/Jaspar(0.710)
6	TAGCCAAACAAA	0.025	2.24%	0.01%	POL004.1_CCAAT-box/Jaspar(0.675)
7	GTTGTGTTTATT	0.025	3.83%	0.17%	MF0005.1_Forkhead_class/Jaspar(0.758)
8	CAAAGAAACG	0.043	7.03%	0.98%	PB0056.1_Rfxdc2_1/Jaspar(0.732)
9	GCATGAAAGTC	0.025	3.19%	0.10%	Brn2(POU,Homeobox)/NPC-Brn2-ChIP-Seq(GSE35496)/Homer(0.665)
10	TATTTATAGCTC	0.035	2.88%	0.07%	MEF2A/MA0052.3/Jaspar(0.815)
11	GCCCACAACCTA	0.036	1.60%	0.00%	KLF4/MA0039.3/Jaspar(0.711)

Supplementary Figure 3. HOMER transcription factor motif analysis. (**A**) Analysis for genes upregulated with senescence comparing last passage to passage 5 shows binding sites for pro-inflammatory transcription factors. (**B**) Analysis for genes downregulated with senescence comparing last passage to passage 5 includes Smad3 binding site and two TATA-box sequences. (**C**) Analysis for genes downregulated comparing passage 10 to passage 5. (FDR=0.05, showing only the best match for each motif).

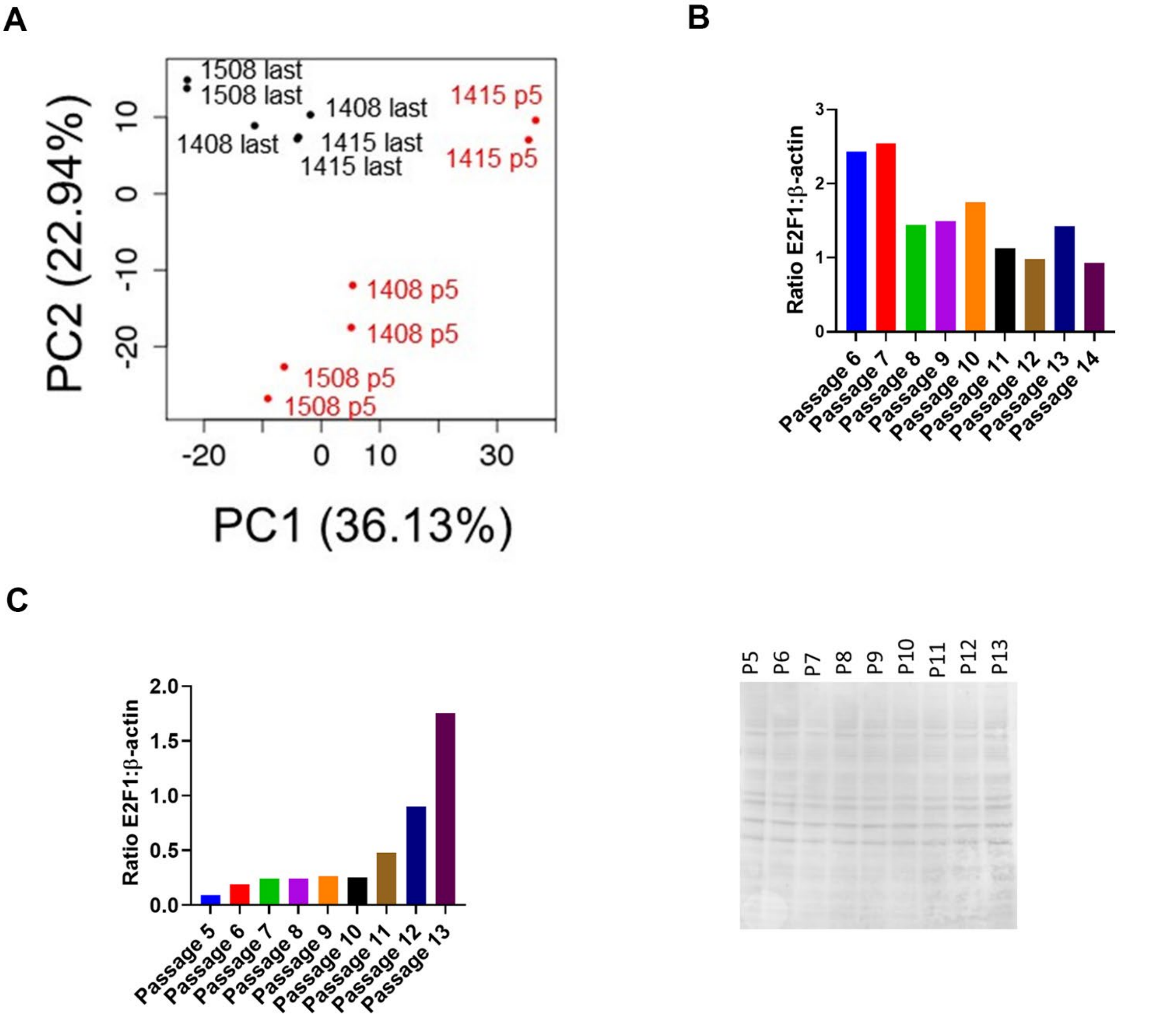

Supplementary Figure 4. Unbiased RNA-seq analysis. (**A**) Principal component analysis plotting passage 5 and last passage for each replicate. (**B**) ImageJ quantification readings of E2F1 normalized to β-actin (Donor 1408). Similar results were obtained for normalization to total protein in Ponceau S stain (Supplementary Figure 1F). (**C**) ImageJ quantification readings of E2F1 normalized to β-actin (Donor 1415). Similar results were obtained for normalization to total protein in Ponceau S stain for total protein (right).

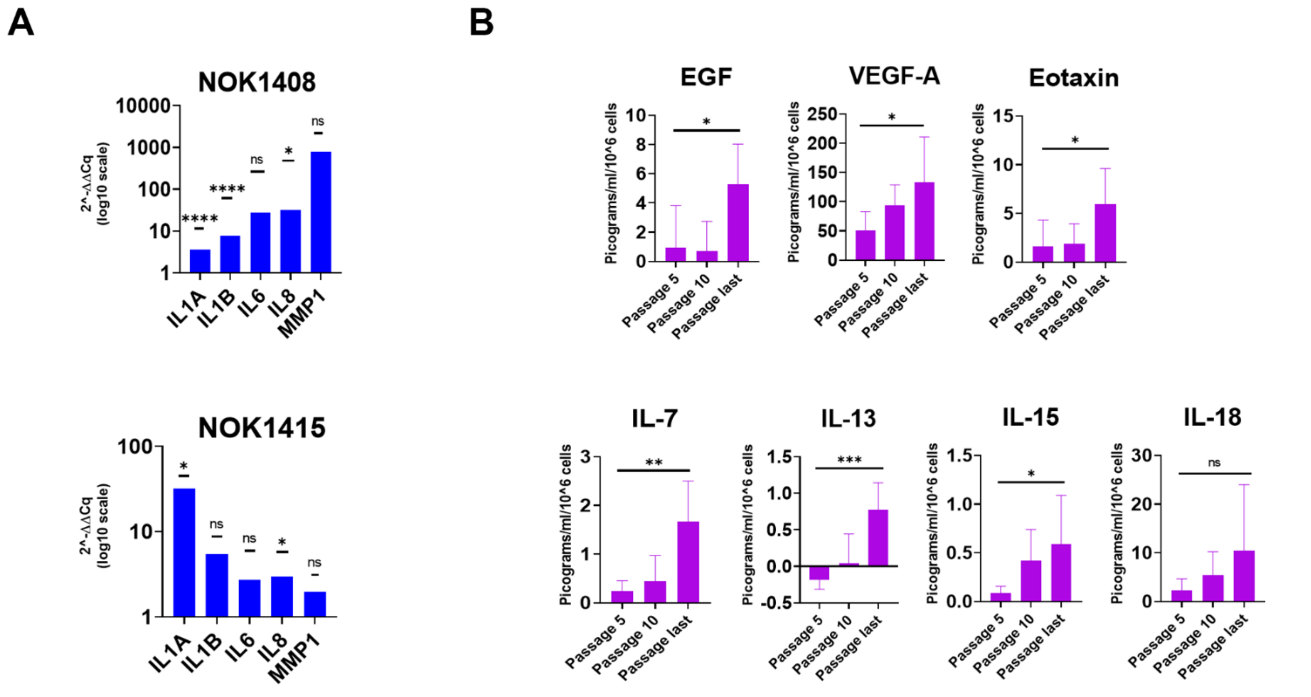

Supplementary Figure 5. SASP components. (**A**) RT-qPCR results for five selected SASP elements. Y-axis is $2^{-\Delta\Delta Cq}$ on a log10 scale. Passage 5 and last passage were normalized to GAPDH (ΔCq) and the last passage was then normalized to passage 5 (ΔΔCq). (Top, two replicates from Donor 1408; bottom, two replicates from Donor 1415). Large difference between female (1408) and male donor (1415) for *MMP1* is consistent with RNA-seq data. Y-axis scales differ. (Significance determined comparing last passage to passage 5, after normalization to GAPDH, using two-tailed t-test). (**B**) Protein levels of other SASP elements in conditioned medium. Determined and normalized as in Figure 4C (Mean ± SD). Y-axis scales differ. (Significance determined by t-test). *p<0.05, **p<0.001, and ****p<0.0001.

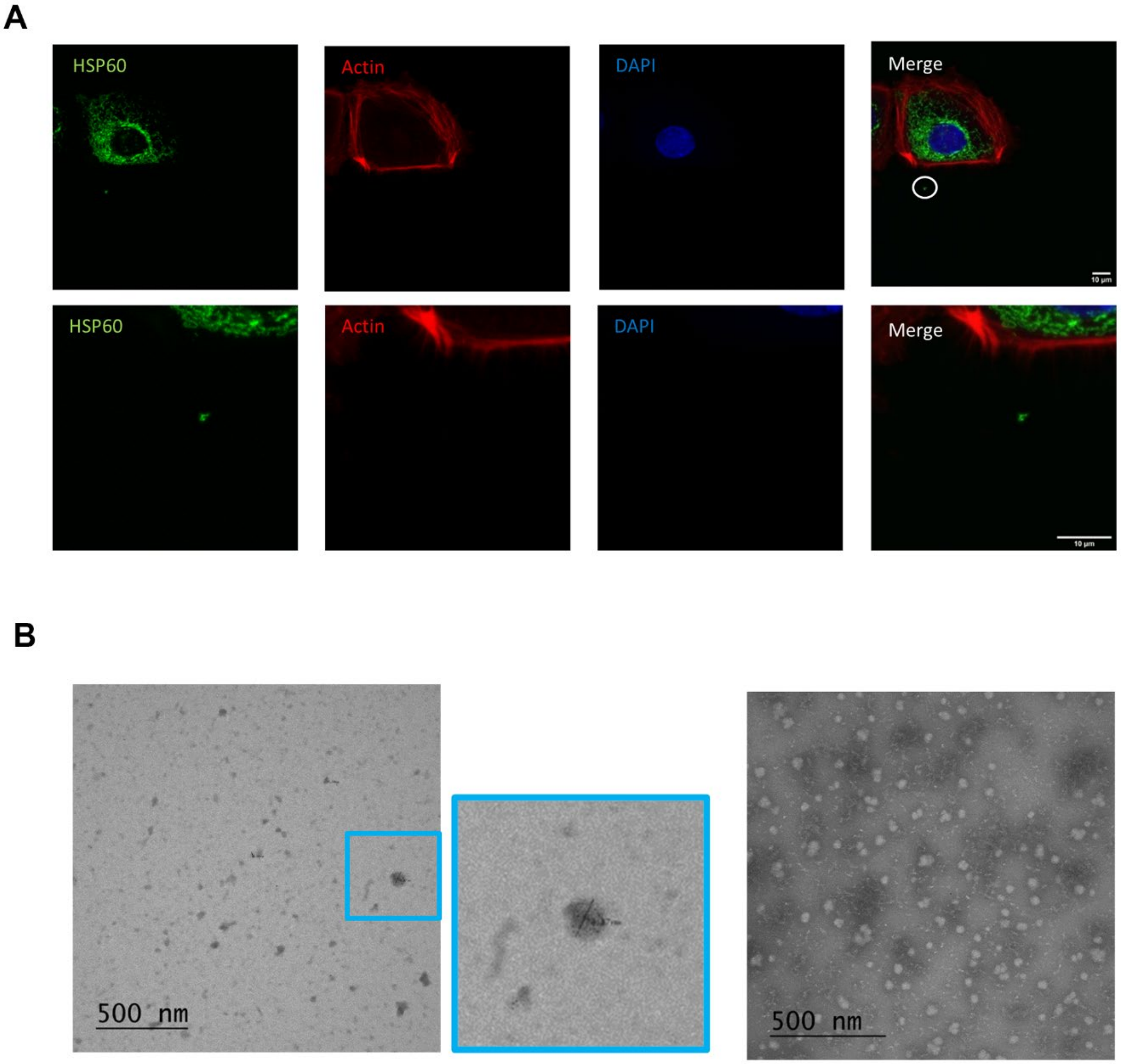

Supplementary Figure 6. HSP60 microscopy. (**A**) IF staining of NOKs for HSP60 and actin. Representative example of many small bodies observed that were positive for HSP60 and outside cell boundary. (Top row, 63x objective and zoom = 1; bottom row, same cell and EV at 63x objective and zoom = 3). (**B**) TEM shows vesicles from EV pellet bearing the positive control exosomal marker CD81 (left, and 3x enlargement center). Negative control (secondary antibody and beads, no primary antibody) shows no labeling of vesicles (right).
