# Supplementary material for: Insights into epithelial cell senescence from transcriptome and secretome analysis of human oral keratinocytes
Source: Aging (Albany NY). 2021 Feb 12;13(4):4747–77. doi: 10.18632/aging.202658 (PMC7950289; doi:10.18632/aging.202658)
Supplement: Supplementary Table 7 [file aging-13-202658-s012.pdf]

SUPPLEMENTARY TABLE

Supplementary Table 7. Primer sequences for RT-qPCR and ddPCR.

ILlα FP	AGTGCTGCTGAAGGAGATGCCTGA
ILlα RP	CCCCTGCCAAGCACACCCAGTA
IL1β FP	TGCACGCTCCGGGACTCACA
IL1 β RP	CATGGAGAACACCACTTGTTGCTCC
IL6 FP	CCAGGAGCCCAGCTATGAAC
IL6 RP	CCCAGGGAGAAGGCAACTG
IL8 FP	GAGTGGACCACACTGCGCCA
IL8 RP	TCCACAACCCTCTGCACCCAGT
MMP1 FP	ATCGGCCCACAAACCCCAAA
MMP1 RP	TGGCAGTTGTGGCCAGAAAACA
ND1 FP	CCCTAAAACCCGCCACATCT
ND1 RP	GAGCGATGGTGAGAGCTAAGGT
ND1 probe	CCATCACCCTCTACATCACCGCCC
